# Supplementary material for: Tyrosine phosphorylation regulates RIPK1 activity to limit cell death and inflammation
Source: Nat Commun. 2022 Nov 3;13:6603. doi: 10.1038/s41467-022-34080-4 (PMC9632600; doi:10.1038/s41467-022-34080-4)
Supplement: Supplementary file 1 — Supplementary Information [file 41467_2022_34080_MOESM1_ESM.pdf]

Tu et al, Tyrosine phosphorylation regulates RIPK1 activity to limit cell death and inflammation

Supplementary Information:

Supplementary Figures 1, 2, 3, 4, 5, 6, 7, 8.

Supplementary Tables 1, 2, 3, 4, 5.

Supplementary Figures

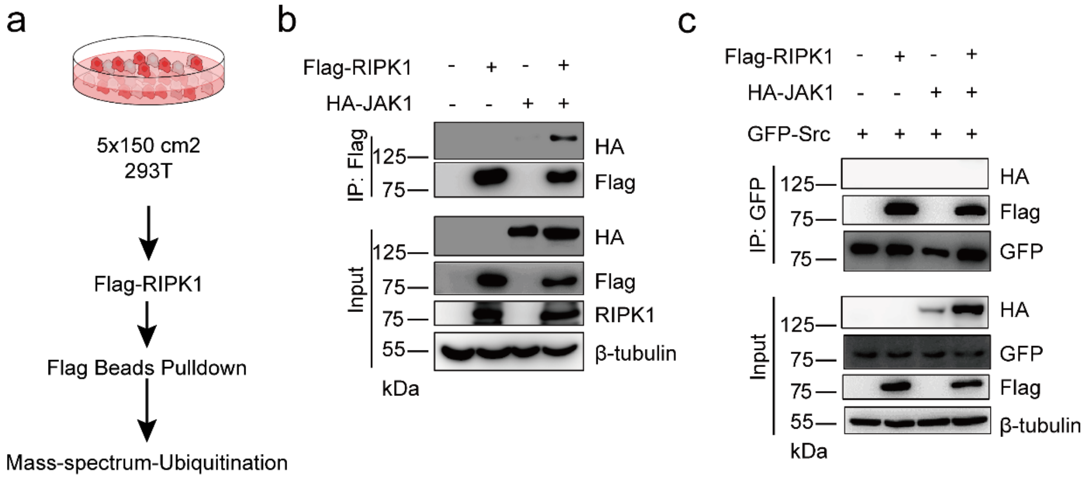

Supplementary Figure 1: Identification of tyrosine phosphorylation as potential regulation of RIPK1 activity

a Schematic overview of mass spectrometry analysis-mediated identification of RIPK1 interacting proteins in 293T cells. PSM: peptide-spectrum match. **b** RIPK1 KO HEK293T cells co-transfected with Flag-RIPK1 and HA-JAK1 for 24h. Cell lysates were immunoprecipitated with anti-Flag-Protein A/G agarose and analyzed by immunoblotting with the indicated antibodies. **c** HEK293T cells co-transfected with Flag-RIPK1, HA-JAK1 and GFP-SRC for 24h. Cell lysates were immunoprecipitated with anti-GFP reesin and analyzed by immunoblotting with the indicated antibodies.

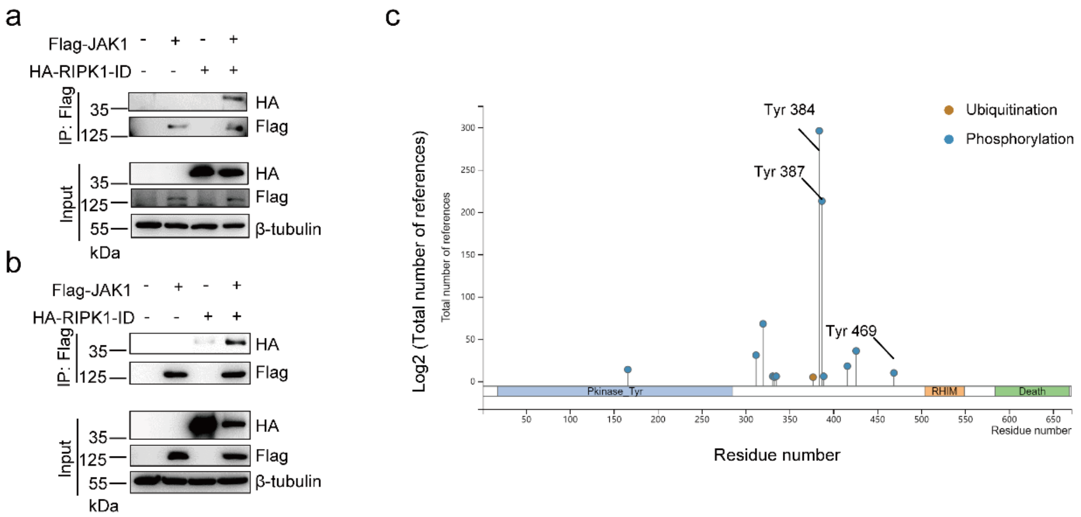

Supplementary Figure 2: Y384 is a major tyrosine phosphorylation site of RIPK1
a, b WT (**a**) or RIPK1 KO (**b**) HEK293T cells co-transfected with HA-RIPK1-ID and Flag-JAK1 for 24h. Cell lysates were immunoprecipitated with anti-Flag-Protein A/G agarose and analyzed by immunoblotting with the indicated antibodies. **c** Schematic of RIPK1 phosphorylation sites from the PhosphoSitePlus database. HTP, high-throughput papers.

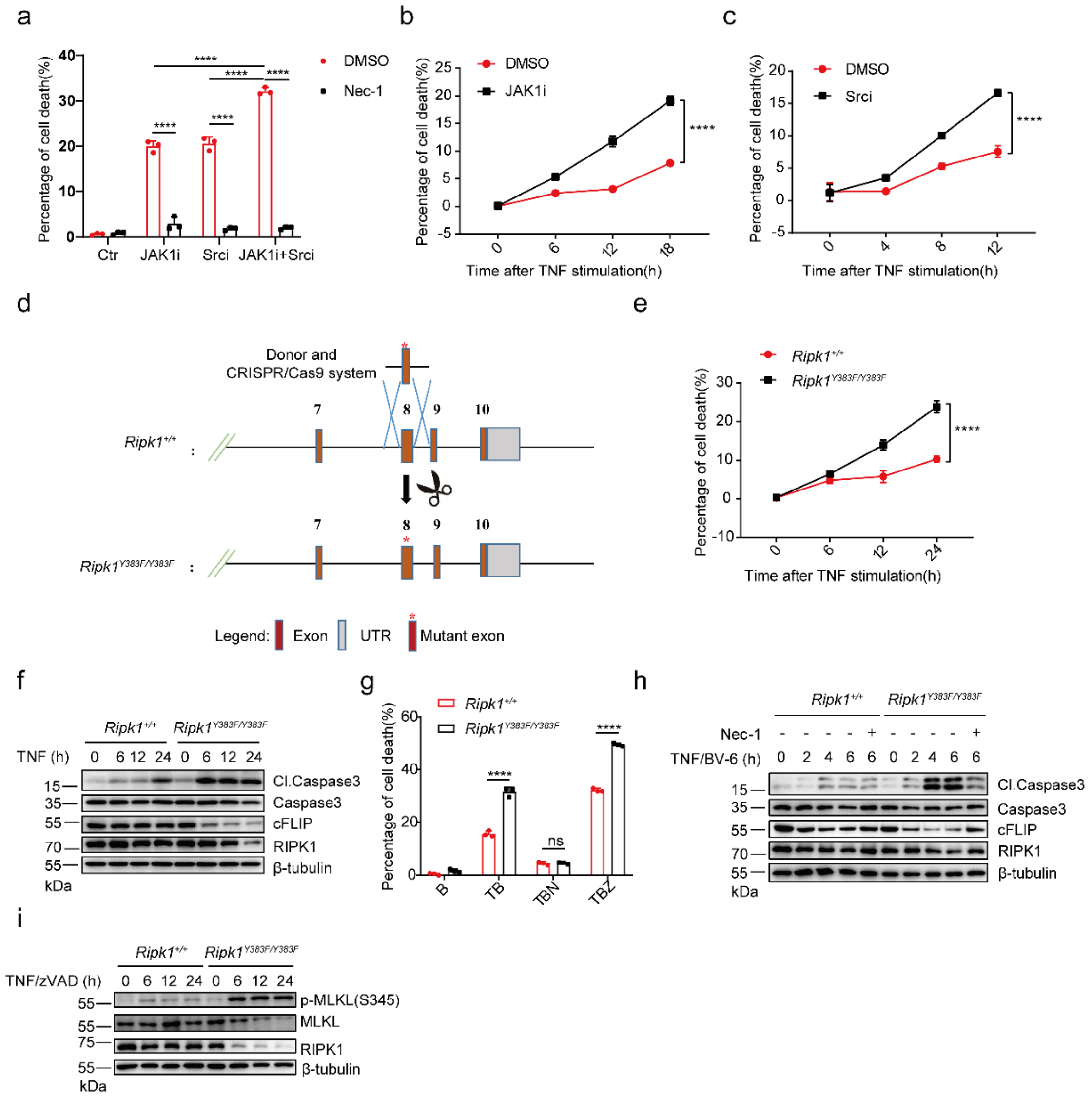

Supplementary Figure 3: Loss of tyrosine phosphorylation of RIPK1 promotes TNF-induced apoptosis and necroptosis

a Primary WT BMDMs were stimulated by TNF/BV-6 with or without Nec-1 (RIPK1 inhibitor), JAK1 and SRC inhibitors for 24h. Cell death were measured by SytoxGreen positivity. **b, c** Immortalized WT MEFs were stimulated by TNF with or without JAK1 (**b**) or SRC (**c**) inhibition treatment for indicated time points and cell death were measured by SytoxGreen positivity. **d** Schematic overview of strategy to generate *Ripk1*$^{Y383F/Y383F}$ mice by CRISPR-Cas9 technology. **e, f** *Ripk1*$^{+/+}$ and *Ripk1*$^{Y383F/Y383F}$ immortalized MEFs were stimulated by TNF for indicated time points. Whole-cell lysates were collected for western blotting (**f**) and cell death were measured by SytoxGreen positivity (**e**). **g** *Ripk1*$^{+/+}$ and *Ripk1*$^{Y383F/Y383F}$ immortalized MEFs were treated by different stimulators for 6h. Cell death were measured by SytoxGreen positivity. T: TNF; B: BV-6; Z: zVAD.fmk; N: Necrostatin-1. **h, i** *Ripk1*$^{+/+}$ and *Ripk1*$^{Y383F/Y383F}$ immortalized MEFs were stimulated by TNF/BV-6 (**h**) and TNF/zVAD (**i**) for indicated time points and whole-cell lysates were collected for western blotting. TNF: 100 ng/ml (**b-c, e-f, i**) and 10 ng/ml (**a, g-h**); BV-6: 2.5 uM; zVAD.fmk: 20 μM; Necrostatin-1: 10 μM; JAK1 inhibitor: 10μM; Src inhibitor: 10μM. In **a-c**, **e** and **g**, data

are represented as mean ± SEM (n = 3 independent cell samples). Statistical significance was determined using a two-tailed unpaired t test. n.s., $p > 0.05$; **** $p < 0.0001$.

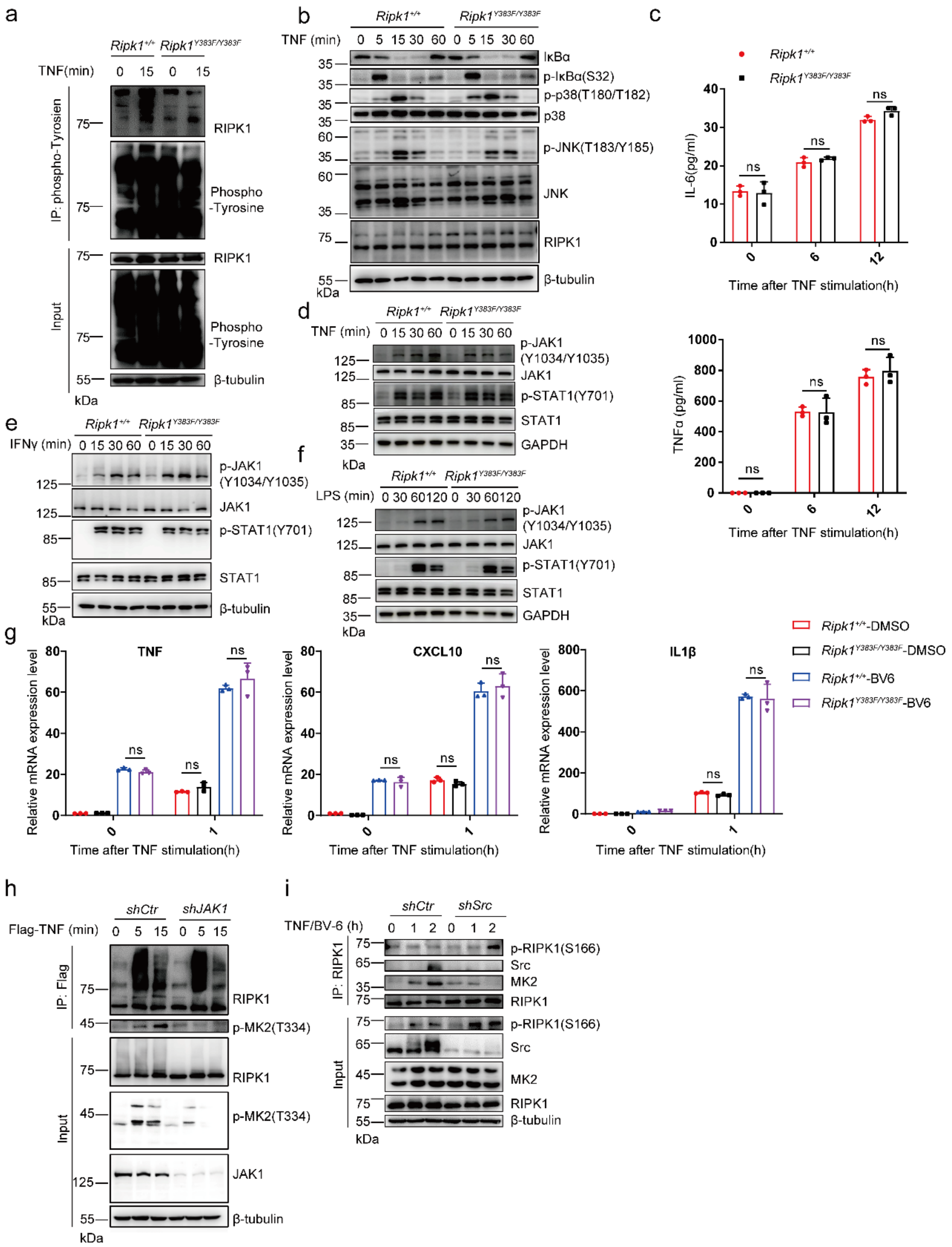

Supplementary Figure 4: Tyrosine phosphorylation of RIPK1 is essential for limiting RIPK1 kinase activity

a *Ripk1*$^{+/+}$ and *Ripk1*$^{Y383F/Y383F}$ immortalized MEFs were stimulated with TNF (10 ng/ml) for indicated time points. Whole-cell lysates were immunoprecipitated with anti-phospho-tyrosine antibody for western blotting with indicated antibodies. **b, d-f** *Ripk1*$^{+/+}$ and *Ripk1*$^{Y383F/Y383F}$ primary BMDMs were stimulated with TNF (**b, d**: 10 ng/ml), IFNγ (**e**: 10 ng/ml) and LPS (**f**: 10 ng/ml) for indicated time points. Whole-cell lysates were collected for western blotting with indicated antibodies. **c** Primary *Ripk1*$^{+/+}$ and *Ripk1*$^{Y383F/Y383F}$ BMDMs were stimulated with TNF (10 ng/ml) at different

time point. The expression level of inflammatory IL6 and TNF were measured by ELISA. **g** Primary *Ripk1*$^{+/+}$ and *Ripk1*$^{Y383F/Y383F}$ BMDMs were stimulated with TNF (10 ng/ml) at indicated time point with or without BV-6 (2.5 uM) pretreatment. The transcriptional and expression level of inflammatory NF-κB target genes were measured by qPCR. **h, i** Immortalized WT MEFs with or without knockdown of JAK1 (**h**) or Src (**i**) were treated with Flag-TNF (100 ng/ml) (**h**) or TNF (10 ng/ml)/BV-6 (2.5 uM) (**i**) for indicated time points and whole-cell lysates were immunoprecipitated using anti-Flag resins (**h**) or anti-RIPK1 (**i**) antibody for western blotting with indicated antibodies. Data are represented as mean ± SEM. Statistical significance was determined using a two-tailed unpaired t test. n.s., $p > 0.05$.

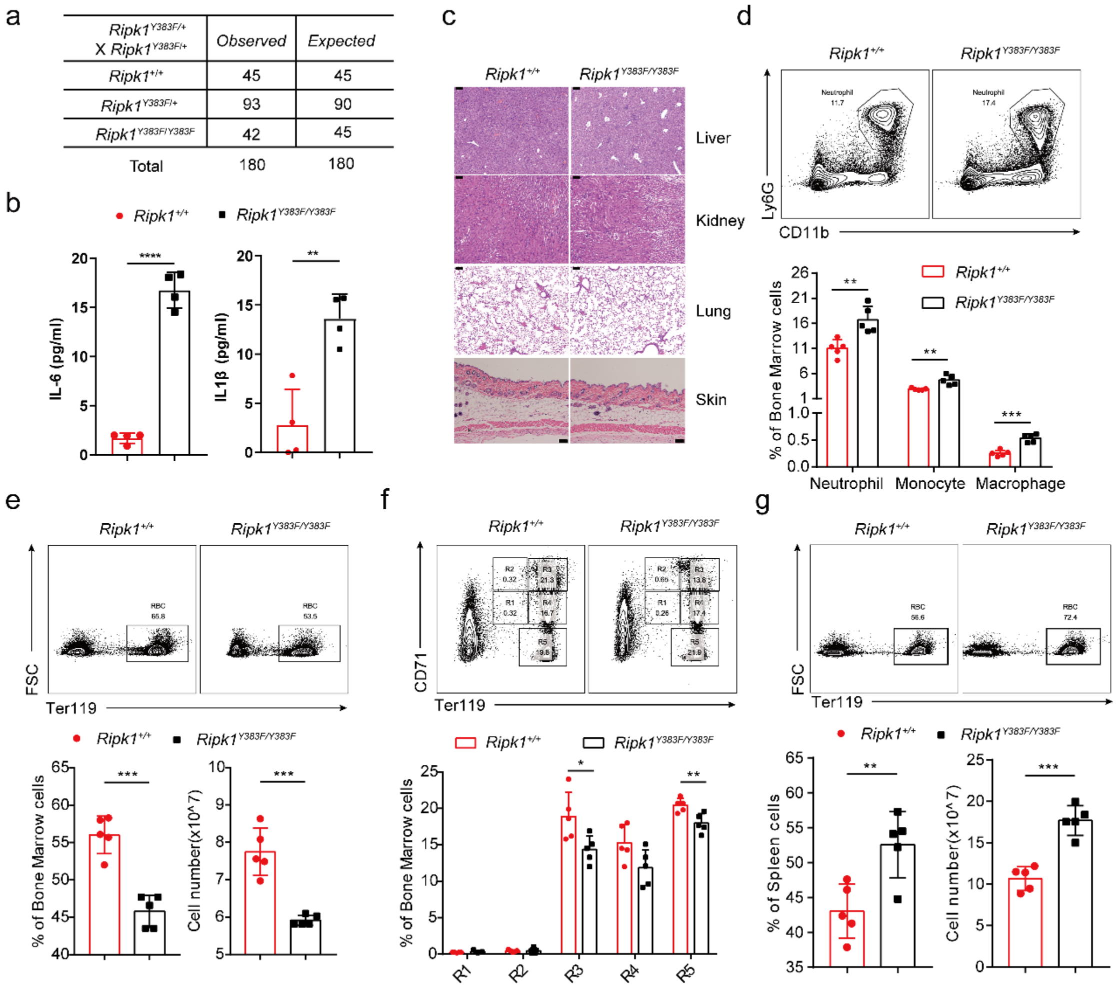

Supplementary Figure 5: RIPK1 Y383F mice develop systemic inflammation and emergency hematopoiesis

a Quantification of genotypes of offspring mice from intercrosses of *Ripk1*$^{Y383F/+}$ mice. **b** The level of IL6 and IL1β in serum of *Ripk1*$^{+/+}$ (n = 4) and *Ripk1*$^{Y383F/Y383F}$ (n = 4) mice at age of 8 weeks. **c** Representative images of H&E staining of liver, kidney, lung and skin tissue from *Ripk1*$^{+/+}$ and *Ripk1*$^{Y383F/Y383F}$ mice at age of 8 weeks (Scale bar, 100 μm). **d** Flow cytometry and statistical analysis of neutrophils, monocytes and macrophages in the bone marrow from *Ripk1*$^{+/+}$ (n = 5) and *Ripk1*$^{Y383F/Y383F}$ (n = 5) mice at age of 8 weeks. **e-g** Flow cytometry and statistical analysis of erythroid lineage Ter119^{+} cells (**e, g**) and different erythroblast populations (**f**) in the bone marrow (**e, f**) and spleen (**g**) from *Ripk1*$^{+/+}$ (n = 5) and *Ripk1*$^{Y383F/Y383F}$ (n = 5) mice at age of 8 weeks. R1 contains immature red blood cell progenitors, including primitive and later-stage erythroid progenitor cells (erythroid burst-forming unit (BFU-E) and colony-forming unit (CFU-E), respectively); R2 comprises mainly pro-erythroblasts and early basophilic erythroblasts; R3 contains both early and late basophilic erythroblasts; R4 is composed of chromatophilic and orthochromatophilic erythroblasts; R5 consists of late orthochromatophilic erythroblasts and reticulocytes. Data are represented as mean ± SEM. Statistical significance was determined using a two-tailed unpaired t test. $*p < 0.05$; $**p < 0.01$; $***p < 0.001$; $****p < 0.0001$.

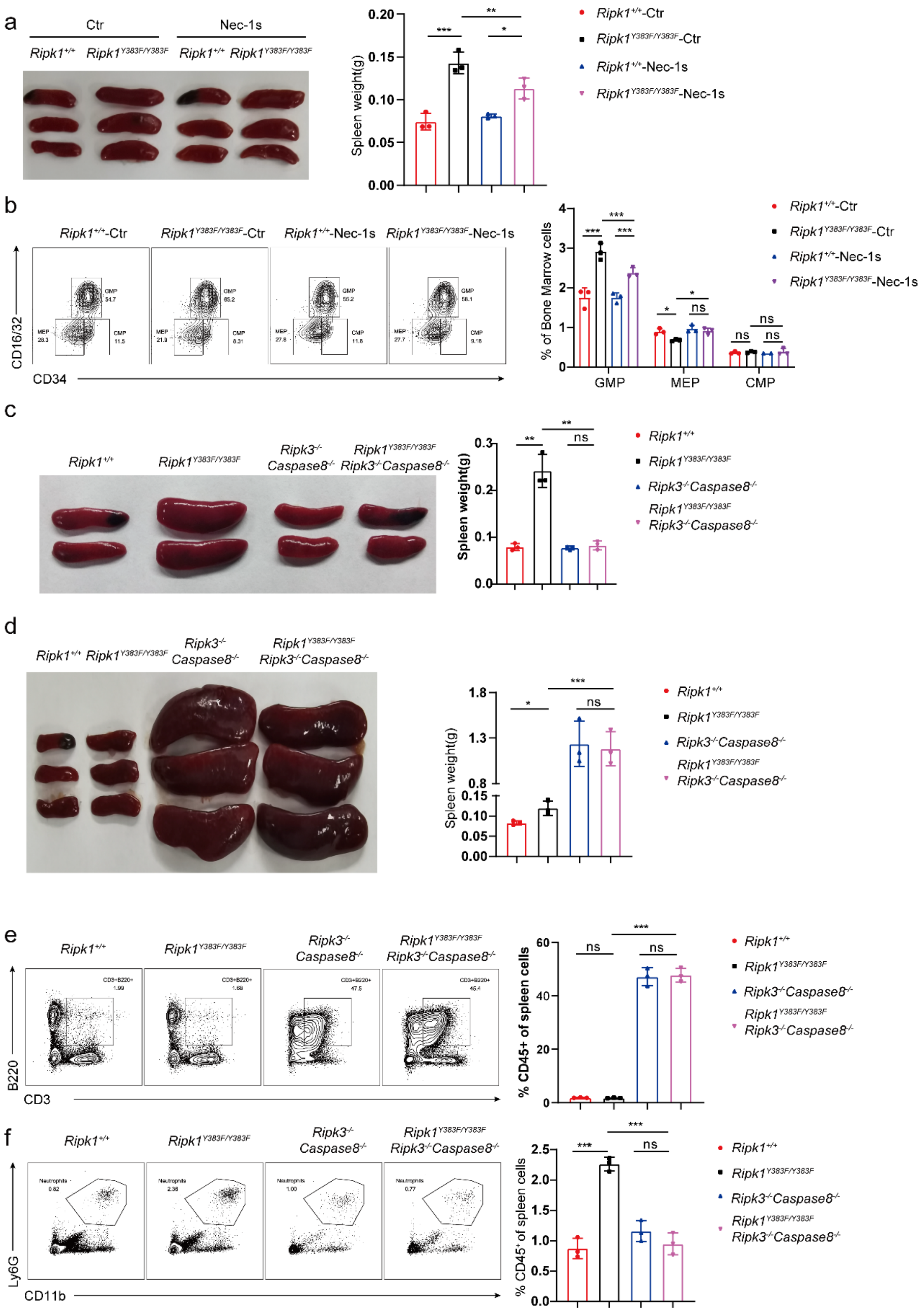

Supplementary Figure 6: TNF-induced cell death is responsible for systemic inflammation and emergency hematopoiesis in RIPK1 Y383F mice

a, b *Ripk1*$^{+/+}$ and *Ripk1*$^{Y383F/Y383F}$ mice were treated with Nec-1s (3mg/kg) every one day after 3 weeks old (n = 3). After 8 weeks, Representative images and weight of skin tissue (**a**) and neutrophils (CD11b^{+}Ly6G^{+}) (**b**) in the spleen were analyzed. **c, d** Representative images and statistical analysis of spleen tissue from *Ripk1*$^{+/+}$,

Ripk1$^{Y383F/Y383F}$, *Ripk3*$^{-/-}$*Caspase8*$^{-/-}$ and *Ripk1*$^{Y383F/Y383F}$ *Ripk3*$^{-/-}$*Caspase8*$^{-/-}$ mice at age of 8 (**c**) and 16 (**d**) weeks (n = 3). **e, f** Flow cytometry and statistical analysis for CD3+B220+ lymphocytes (**e**) and neutrophils (CD11b^{+}Ly6G^{+}) (**f**) in the spleen from *Ripk1*$^{+/+}$, *Ripk1*$^{Y383F/Y383F}$, *Ripk3*$^{-/-}$*Caspase8*$^{-/-}$ and *Ripk1*$^{Y383F/Y383F}$ *Ripk3*$^{-/-}$*Caspase8*$^{-/-}$ mice at age of 16 weeks (n = 3). Data are represented as mean ± SEM. Statistical significance was determined using a two-tailed unpaired t test. n.s., $p > 0.05$; $*p < 0.05$; $**p < 0.01$; $***p < 0.001$.

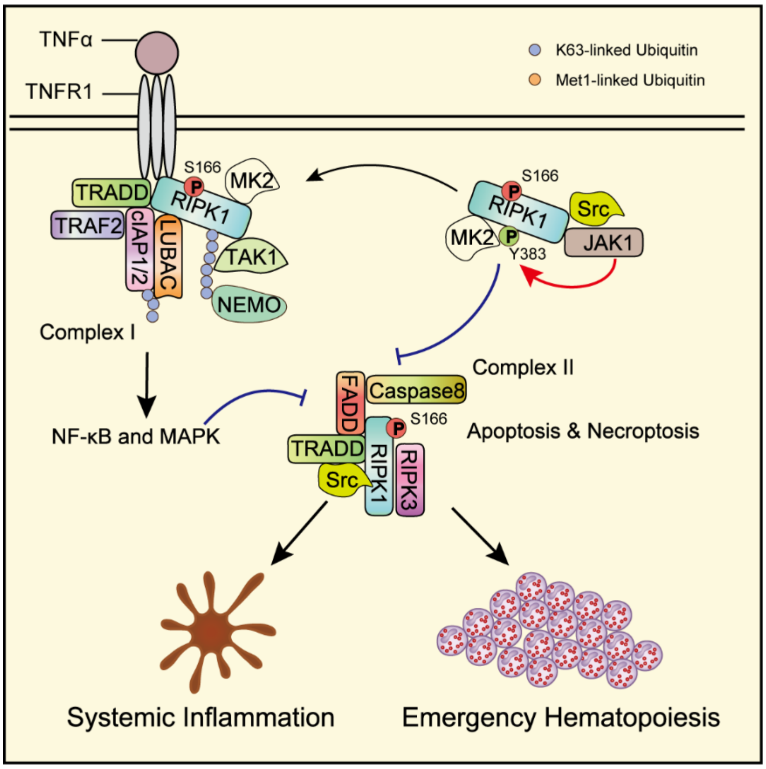

Supplementary Figure 7: Proposed model that tyrosine phosphorylation of RIPK1 on Y383 regulates RIPK1 kinase activity to prevent cell death and inflammation

Up, ligation of TNF on TNFR1 induces formation of TNF-RSC, and subsequent phosphorylation and ubiquitination of RIPK1 to activate NF-κB and MAPK-dependent survival genes. These post-translational modifications of RIPK1 could stabilize TNF-RSC and further inhibit cytotoxic activity of RIPK1. Herein, we identify non-receptor tyrosine kinases JAK1 and SRC could phosphorylate RIPK1 on Y383 to recruit and activate downstream kinase MK2. **Down**, deficiency of tyrosine phosphorylation of RIPK1 by Y383F mutation activates RIPK1 kinase partially though blocking MK2 activation, which leads to enhanced TNF-induced apoptosis and necroptosis and systemic inflammation and emergency hematopoiesis in *Ripk1*$^{Y383F/Y383F}$ mice.

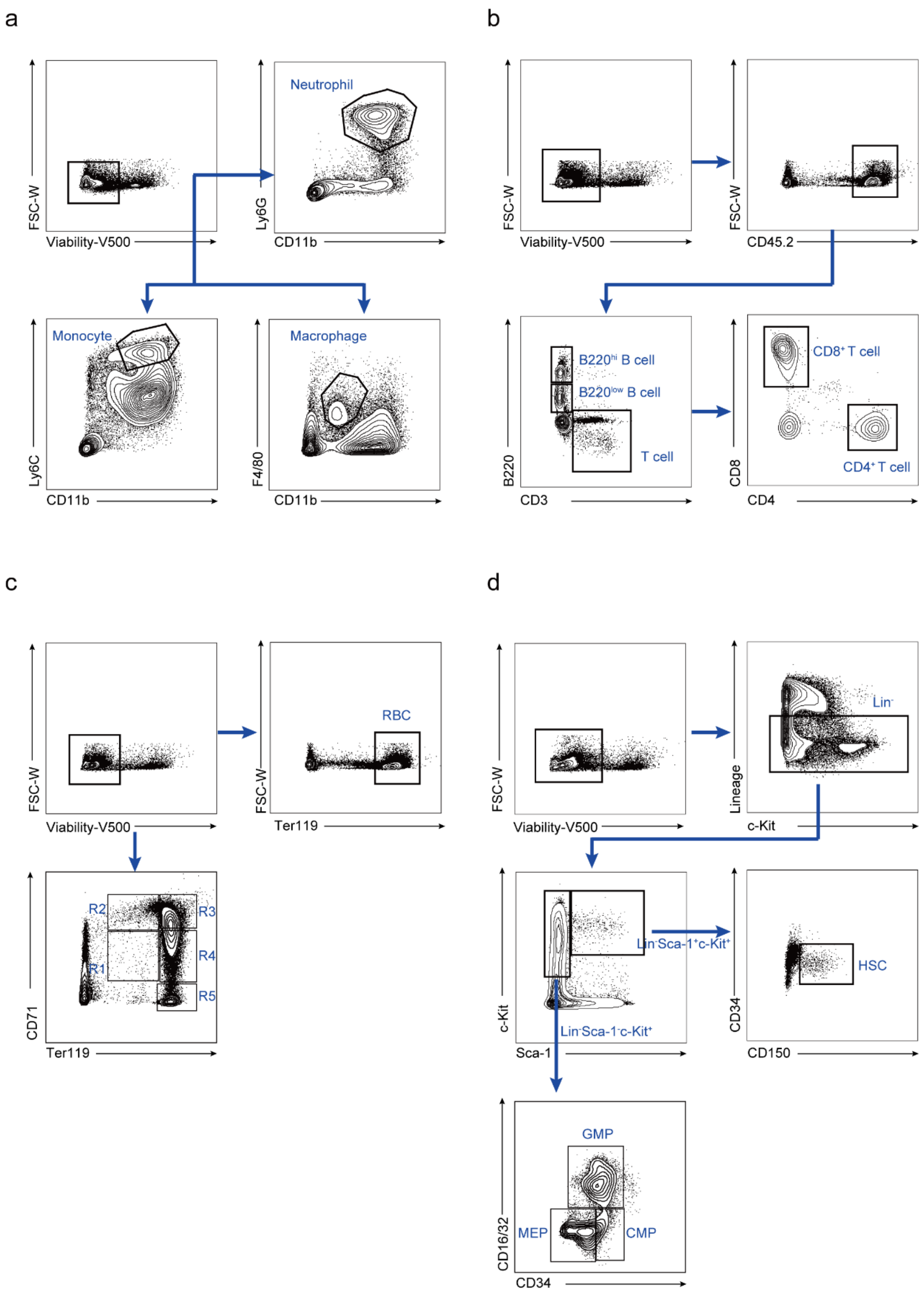

Supplementary Figure 8: Hematopoietic gating strategy

a, For characterization of myeloid cell in spleen and bone marrow, the living cell fractions gated from preliminary FSC/SSC gates could be further divided into CD11b$^+$Ly6G$^+$ neutrophils, CD11b$^+$Ly6C$^+$ inflammatory monocytes, CD11b$^+$F4/80$^+$ macrophages. **b**, For characterization of lymphocytes in spleen, bone marrow and thymus, the living cell fractions gated from preliminary FSC/SSC gates could be further

divided into $B220^+$ B cells, $CD3^+$ T cells, $B220^-CD3^+CD4^+$ T cells and $B220^-CD3^+CD8^+$ T cells. **c,** For characterization of red blood cells and erythroblast populations in spleen, bone marrow, the living cell fractions gated from preliminary FSC/SSC gates could be further divided into $Ter119^+$ red blood cells, $Ter119^{int}CD71^{int}$ erythroblast R1, $Ter119^{int}CD71^{high}$ erythroblast R2, $Ter119^{high}CD71^{high}$ erythroblast R3, $Ter119^{high}CD71^{int}$ erythroblast R4 and $Ter119^{high}CD71^{low}$ erythroblast R5 population.
d, For characterization of hematopoietic progenitor populations in bone marrow, the living cell fractions gated from preliminary FSC/SSC gates could be further divided into $Lin^-Sca\text{-}1^+Kit^+$ (LSKs) progenitors, $Lin^-Sca\text{-}1^-Kit^+$ (LKs) progenitors, LSK-$CD150^+CD34^-$ Hematopoietic stem cells, $Lin^-Sca\text{-}1^-Kit^+Fc\gamma R^+CD34^+$(GMPs) progenitors, $Lin^-Sca\text{-}1^-Kit^+Fc\gamma R^-CD34^+$ (CMPs) progenitors and $Lin^-Sca\text{-}1^-Kit^+Fc\gamma R^-CD34^-$ (MEPs) progenitors.

Supplementary Tables

Supplementary Table 1: Identification of RIPK1 interacting proteins via mass spectrum.

RIPK1-Mass-spectrum	Protein	PSM
**Scaffold proteins**	TAB1	32
	TAB2	24
	TRADD	15
	TAB3	1
**Cell death complexes**	CASPASE8	131
	FADD	109
	CASPASE9	3
	cFLIP	2

Supplementary Table 2: Primer sequences for generation of *Ripk1*$^{Y383F/ Y383F}$ mice.

sgRNA	5'-tattccaaaagcatgataggc-3'
Donor	5'-cacaggacgagaatgatcgcagtgtgcaggctaagctgcaagaggaagc aagttttcatgcttttggaatatttgcagagaaacagacaaaaccgcagccaaggca gaatgaggcttacaa-3'

Supplementary Table 3. The primer sequences for genotyping PCR**.**

	Forward Primer (5’--3’)	Reverse Primer (5’--3’)
RIPK1 WT	agctgcaagaggaagccagcta	gtgctgggatcagaatgacc
RIPK1 K376R	agctgcaagaggaagcaagttt	gtgctgggatcagaatgacc

Supplementary Table 4. The primer sequences for qRT-PCR**.**

Gene	Forward Primer (5'--3')	Reverse Primer (5'--3')
Gapdh	aacagcaactcccactcttc	cctgttgctgtagccgtatt
Il1β	gaaatgccaccttttgacagtg	tggatgctctcatcaggacag
Tnf	ctaccttgttgcctcctcttt	gagcagaggttcagtgatgtag
IκBα	tgaaggacgaggagtacgagc	ttcgtggatgattgccaagtg
Cxcl10	ccaagtgctgccgtcattttc	ggctcgcagggatgatttcaa

Supplementary Table 5. The primer sequences for generating MK2 deficient MEFs**.**

	Oligo 1	Oligo 2
MK2 sgRNA1	CACCGcgccatcaccgacgactaca	AAACtgtagtcgtcggtgatggcgC
MK2 sgRNA2	CACCGggcccgacttgacgtggaac	AAACgttccacgtcaagtcgggccC
