# Supplementary material for: Tyrosine phosphorylation regulates RIPK1 activity to limit cell death and inflammation
Source: Nat Commun. 2022 Nov 3;13:6603. doi: 10.1038/s41467-022-34080-4 (PMC9632600; doi:10.1038/s41467-022-34080-4)
Supplement: Supplementary file 3 — Reporting Summary [file 41467_2022_34080_MOESM3_ESM.pdf]

nature portfolio

Corresponding author(s): Xin Lin

Last updated by author(s): Aug 29, 2022

Reporting Summary

Nature Portfolio wishes to improve the reproducibility of the work that we publish. This form provides structure for consistency and transparency in reporting. For further information on Nature Portfolio policies, see our Editorial Policies and the Editorial Policy Checklist.

Statistics

For all statistical analyses, confirm that the following items are present in the figure legend, table legend, main text, or Methods section.

n/a	Confirmed	
☐	☒	The exact sample size (*n*) for each experimental group/condition, given as a discrete number and unit of measurement
☐	☒	A statement on whether measurements were taken from distinct samples or whether the same sample was measured repeatedly
☐	☒	The statistical test(s) used AND whether they are one- or two-sided<br>*Only common tests should be described solely by name; describe more complex techniques in the Methods section.*
☒	☐	A description of all covariates tested
☐	☒	A description of any assumptions or corrections, such as tests of normality and adjustment for multiple comparisons
☐	☒	A full description of the statistical parameters including central tendency (e.g. means) or other basic estimates (e.g. regression coefficient) AND variation (e.g. standard deviation) or associated estimates of uncertainty (e.g. confidence intervals)
☐	☒	For null hypothesis testing, the test statistic (e.g. *F*, *t*, *r*) with confidence intervals, effect sizes, degrees of freedom and *P* value noted<br>*Give P values as exact values whenever suitable.*
☒	☐	For Bayesian analysis, information on the choice of priors and Markov chain Monte Carlo settings
☒	☐	For hierarchical and complex designs, identification of the appropriate level for tests and full reporting of outcomes
☒	☐	Estimates of effect sizes (e.g. Cohen's *d*, Pearson's *r*), indicating how they were calculated

Our web collection on statistics for biologists contains articles on many of the points above.

Software and code

Policy information about availability of computer code

Data collection	FACSAria II cytometer, ABI 7500 Real-Time PCR system
Data analysis	GraphPad Prism, FlowJo,

For manuscripts utilizing custom algorithms or software that are central to the research but not yet described in published literature, software must be made available to editors and reviewers. We strongly encourage code deposition in a community repository (e.g. GitHub). See the Nature Portfolio guidelines for submitting code & software for further information.

Data

Policy information about availability of data

All manuscripts must include a data availability statement. This statement should provide the following information, where applicable:

- Accession codes, unique identifiers, or web links for publicly available datasets
- A description of any restrictions on data availability
- For clinical datasets or third party data, please ensure that the statement adheres to our policy

The authors declare that the data supporting the findings of this study are available within the paper and its Supplementary Information Files. Source data are provided as source data files. The source data of statistical analysis in the figures are provided as a Source Data file 1. The source data of uncropped scans of Western-blots in the figures are provided as a Source Data file 2. Mass-spectrum analysis refer to Uniprot database and the source data are provided as a source data file 3.

Human research participants

Policy information about studies involving human research participants and Sex and Gender in Research.

Reporting on sex and gender	NA
Population characteristics	NA
Recruitment	NA
Ethics oversight	NA

Note that full information on the approval of the study protocol must also be provided in the manuscript.

Field-specific reporting

Please select the one below that is the best fit for your research. If you are not sure, read the appropriate sections before making your selection.

☒ Life sciences ☐ Behavioural & social sciences ☐ Ecological, evolutionary & environmental sciences

For a reference copy of the document with all sections, see nature.com/documents/nr-reporting-summary-flat.pdf

Life sciences study design

All studies must disclose on these points even when the disclosure is negative.

Sample size	The cell samples were collected and performed with at least three independent experiments. The samples from animal were collected from three mice every group and performed with three independent experiments. The human samples were independently collected for at least three times and measured with two duplicates. The exact size was described in the figure or the legend. For animal experiments, we choose same age of control and experiment littermate mice. We measure the weight of body, spleen and other organs. For the cellular experiments, we count the cell number and use the same amount of control and experiment cells to do further experimetns.
Data exclusions	No data were excluded in the analysis in this study.
Replication	All experimental findings described in this study were repeated at least three times and got the consistent results before make relative conclusions.
Randomization	The samples/organisms/participants were randomly allocated into experimental group.
Blinding	All the authors involved in the experiments in this study were blinded to group allocation during data collection and/or analysis.

Reporting for specific materials, systems and methods

We require information from authors about some types of materials, experimental systems and methods used in many studies. Here, indicate whether each material, system or method listed is relevant to your study. If you are not sure if a list item applies to your research, read the appropriate section before selecting a response.

Materials & experimental systems

n/a	Involved in the study	
☐	☒	Antibodies
☐	☒	Eukaryotic cell lines
☒	☐	Palaeontology and archaeology
☐	☒	Animals and other organisms
☒	☐	Clinical data
☒	☐	Dual use research of concern

Methods

n/a	Involved in the study	
☒	☐	ChIP-seq
☐	☒	Flow cytometry
☒	☐	MRI-based neuroimaging

Antibodies

Antibodies used	The following antibodies were used in western blotting experiments: Antibodies against RIPK1 (3493), phospho-RIPK1 (31122), cleaved Caspase3 (9664), Caspase3 (9665), cleaved Caspase8 (9429), Caspase8 (4790), A20 (5630), p-IκBα (2859), p-p38 (9211), p38 (9228), p-JNK (9251), JNK (9252), p-TBK1 (5483), TBK1 (3504), JAK1 (50996), p-JAK1 (74129), STAT1 (14994), p-STAT1 (9167), SRC

(2109), p-MK2 (3007), MK2 (3042), cFLIP (56343) and RelB (4954) were purchased from Cell Signaling Technology; Antibodies against RIPK3 (sc-374639), IκBα (sc-1643), PCNA (sc-56), FADD (sc-6036) and p65 (sc-109) were purchased from Santa Cruz Biotechnology; Antibodies against phospho-MLKL (ab196436) and MLKL (ab67942); Antibodies against β-tubulin (BE0025-100), GFP (BE2002), GAPDH (BE0023), secondary horseradish peroxidase (HRP)-conjugated anti-rabbit (BE0108-100) and anti-mouse antibodies (BE0107-100) were obtained from Easy Bio; Antibody against phosphor-tyrosine (4G10) were purchased from Milllipore; Antibody against Flag (M20008), HA (M20006) were purchased from Abmart. AF700 anti B220 (48-0452-80), V450 anti-CD3 (48-0032-8246-0041-82), Percp-cy5.5 anti-CD4 (46-0041-82), FITC anti-CD8 (11-0081-82), FITC anti-CD11b (11-0112-82), PE anti-F4/80 (12-4801-82), PE anti-c-Kit (12-1171-81), Percp-cy5.5 anti-CD16/32 (45-0161-82) and V500-conjugated Cell viability Dye(65-0866-18) were purchased from eBioscience; Percp-cy5.5 anti-Ly6C (561237), V450 anti-Ly6G (560603), FITC anti-CD34 (553733) antibodies were from BD Bioscience; APC anti-CD71 (113819), PE anti-Ter119 (116207), PB anti-Sca-1 (108120), APC anti-CD150 (115909) and FITC anti-Lineage (133313) antibodies were from BioLegend. Antibodies for western-blotting were used with 500-fold or 1000-fold dilution and for flow cytometry were used with 200-fold dilution.

Validation: All antibodies used in this study were validated accrording to the manufacturer's instruction and all worked well.

Eukaryotic cell lines

Policy information about cell lines and Sex and Gender in Research

Cell line source(s): The source of HEK293T cells were from ATCC, and the source of immortalized MEFs and BMDMs were described in the methods.

Authentication: The HEK293T cells were purchased from ATCC and authenticated by morphology and the vendor, and the immortalized MEFs were authenticated according to the genotypes of embryos.

Mycoplasma contamination: We declare that all cell lines used in this research are mycoplasma negative by experimental detection.

Commonly misidentified lines (See ICLAC register): No commonly misidentified cell lines were used.

Animals and other research organisms

Policy information about studies involving animals; ARRIVE guidelines recommended for reporting animal research, and Sex and Gender in Research

Laboratory animals: Research animals used in this study are RIPK1 Y383F, Tnfr1-/-, Ripk3-/- and Caspase-8-/- mouse lines on a C57BL6/J background. These mice are housed in the specific pathogen-free (SPF) animal facilities (light/dark cycle 10h:14h, temperature 22-26℃, humidity 40%-70%).

Wild animals: The study did not involve wild animals.

Reporting on sex: The study did not involve sex-based analysis.

Field-collected samples: The study did not involve samples collected from the field.

Ethics oversight: All mouse experiments were performed in compliance with institutional guidelines and according to the protocol approved by the Institutional Animal Care and Use Committee of Tsinghua University.

Note that full information on the approval of the study protocol must also be provided in the manuscript.

Flow Cytometry

Plots

Confirm that:

- [x] The axis labels state the marker and fluorochrome used (e.g. CD4-FITC).
- [x] The axis scales are clearly visible. Include numbers along axes only for bottom left plot of group (a 'group' is an analysis of identical markers).
- [x] All plots are contour plots with outliers or pseudocolor plots.
- [x] A numerical value for number of cells or percentage (with statistics) is provided.

Methodology

Sample preparation: For mice samples, the spleens from mice were perfused and digested into single-cell suspensions. After RBC lysis buffer treatment, the whole cells were washed with PBS and then stained with corresponding fluorescent antibodies and left at 4℃ until use. All mouse experiments were performed in compliance with institutional guidelines and according to the protocol approved by the Institutional Animal Care and Use Committee of Tsinghua University.

Instrument: For cell analysis, LSRFortessa (BD Biosciences) was used.

Software	Flow cytometry data were analyzed with FlowJo (Tree Star).
Cell population abundance	Without using sorting strategy.
Gating strategy	In the spleen or bone marrow of mice, the living cell fractions gated from preliminary FSC/SSC gates could be further divided into four cell types: CD4+ T cells, CD8+ T cells, CD19+B220+ B cells, CD11b+Ly6G+ neutrophils, CD11b+F4/80+ macrophage, CD11b+Ly6C+ monocyte, Ter119+ red blood cells, Ter119intCD71int erythroblast R1, Ter119intCD71high erythroblast R2, Ter119highCD71high erythroblast R3, Ter119highCD71int erythroblast R4, Ter119highCD71low erythroblast R5, Lin-Sca-1+Kit+ (LSKs) progenitors, Lin-Sca-1-Kit+ (LKs) progenitors, LSK-CD150+CD34- Hematopoietic stem cells, Lin-Sca-1-Kit+FcγR+CD34+(GMPs) progenitors, Lin-Sca-1-Kit+FcγR-CD34+ (CMPs) progenitors and Lin-Sca-1-Kit+FcγR-CD34- (MEPs) progenitors. All this is shown in supplementary Figures and information.

☒ Tick this box to confirm that a figure exemplifying the gating strategy is provided in the Supplementary Information.
