# Supplementary material for: Tyrosine phosphorylation regulates RIPK1 activity to limit cell death and inflammation
Source: Nat Commun. 2022 Nov 3;13:6603. doi: 10.1038/s41467-022-34080-4 (PMC9632600; doi:10.1038/s41467-022-34080-4)

Figure 1a

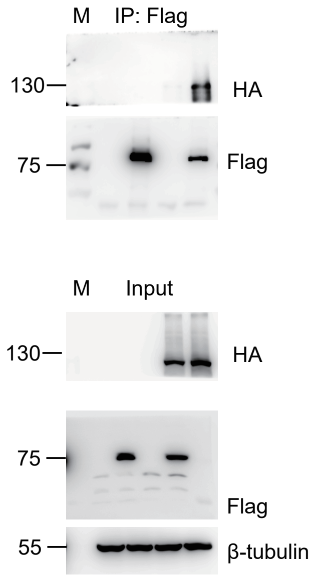

Figure 1b

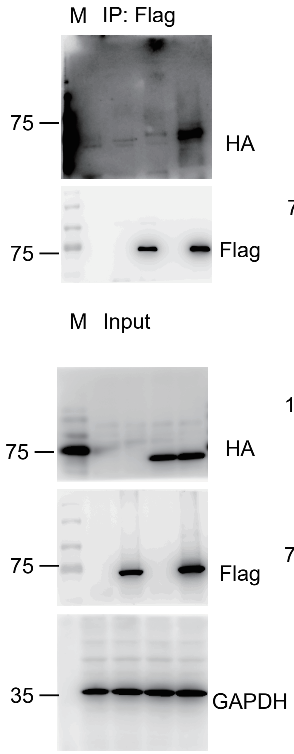

Figure 1c

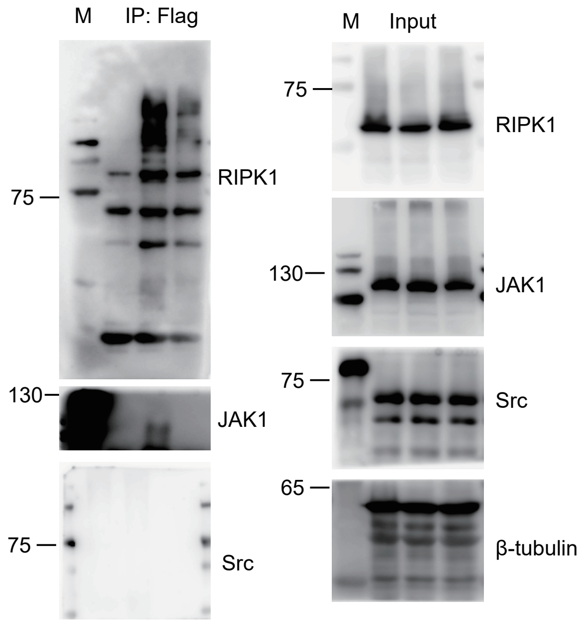

Figure 1d

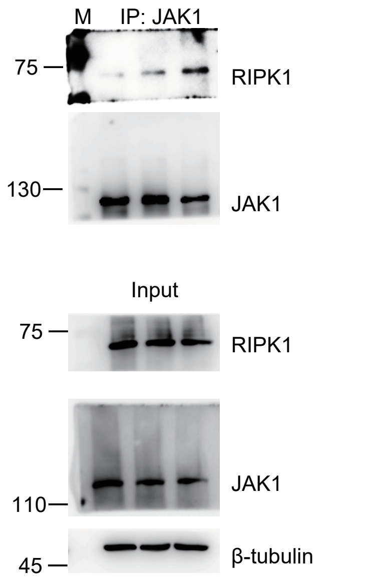

Figure 1e

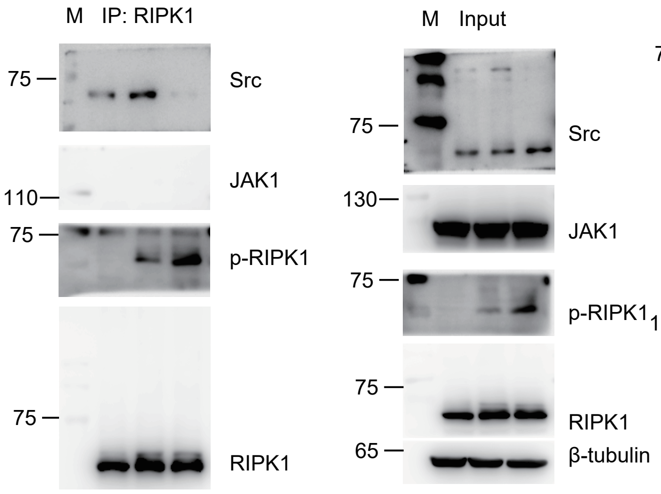

Figure 1f

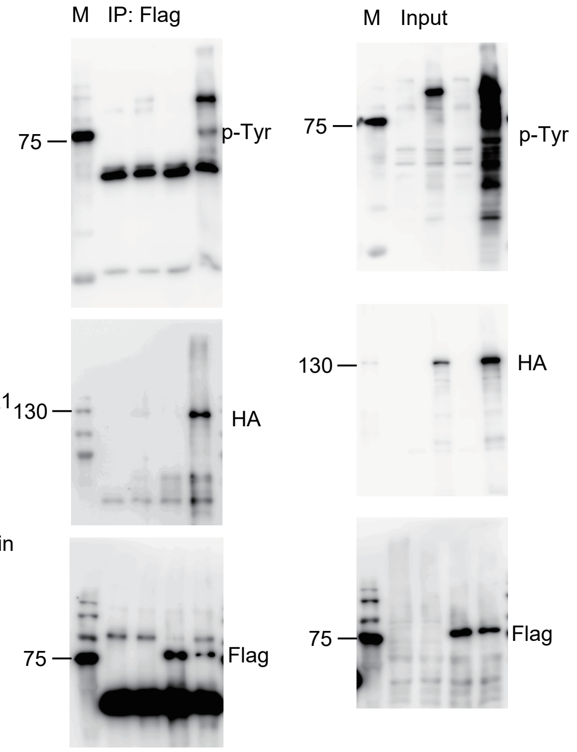

Figure 1h

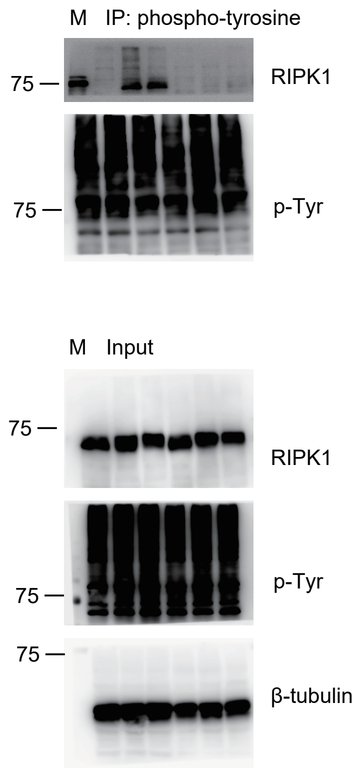

Figure 1g

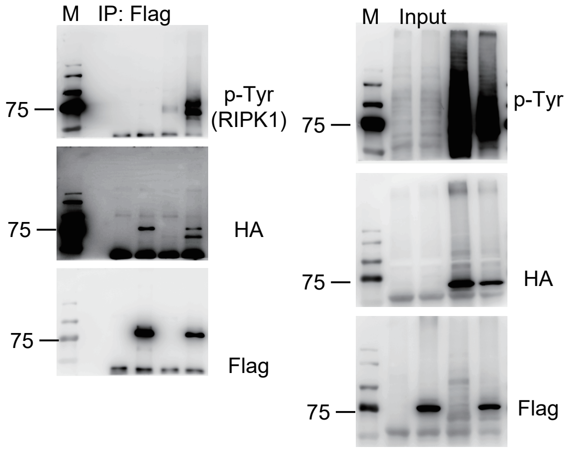

Figure 2b

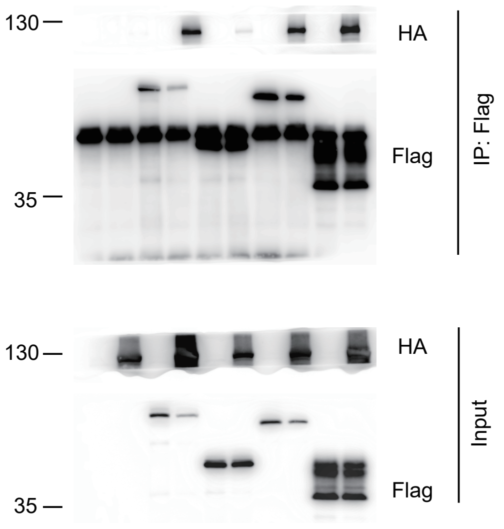

Figure 2c

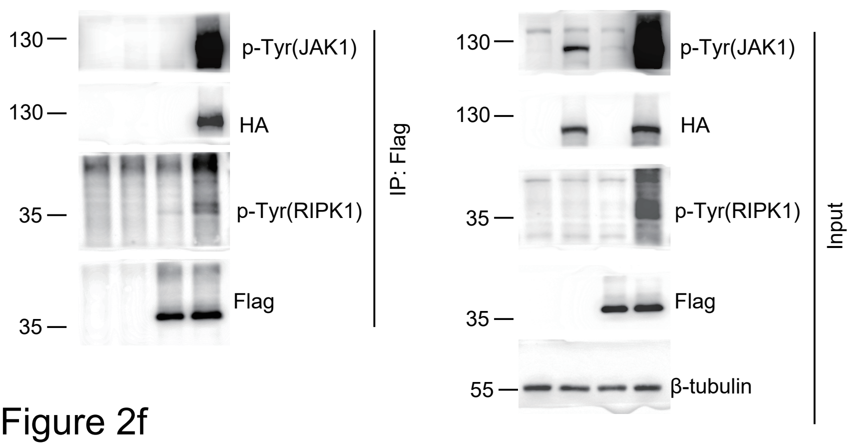

Figure 2d

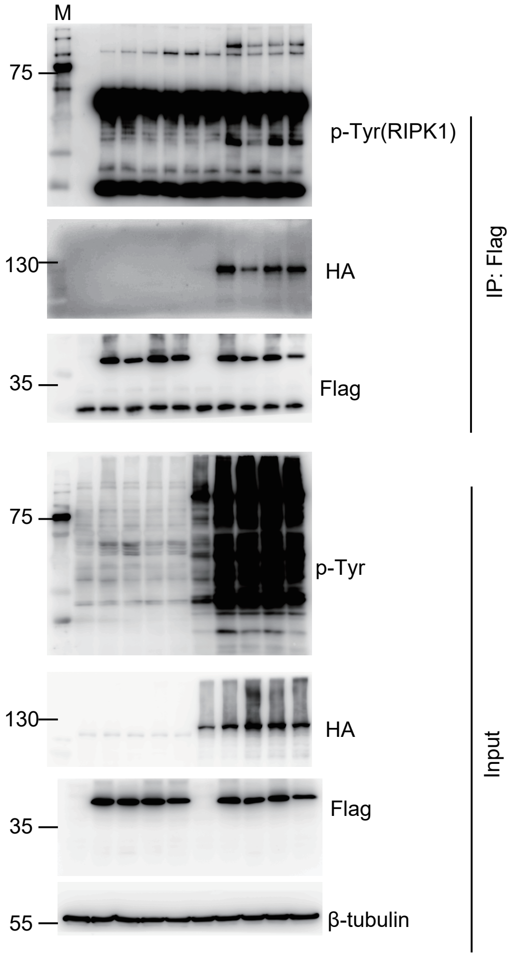

Figure 2f

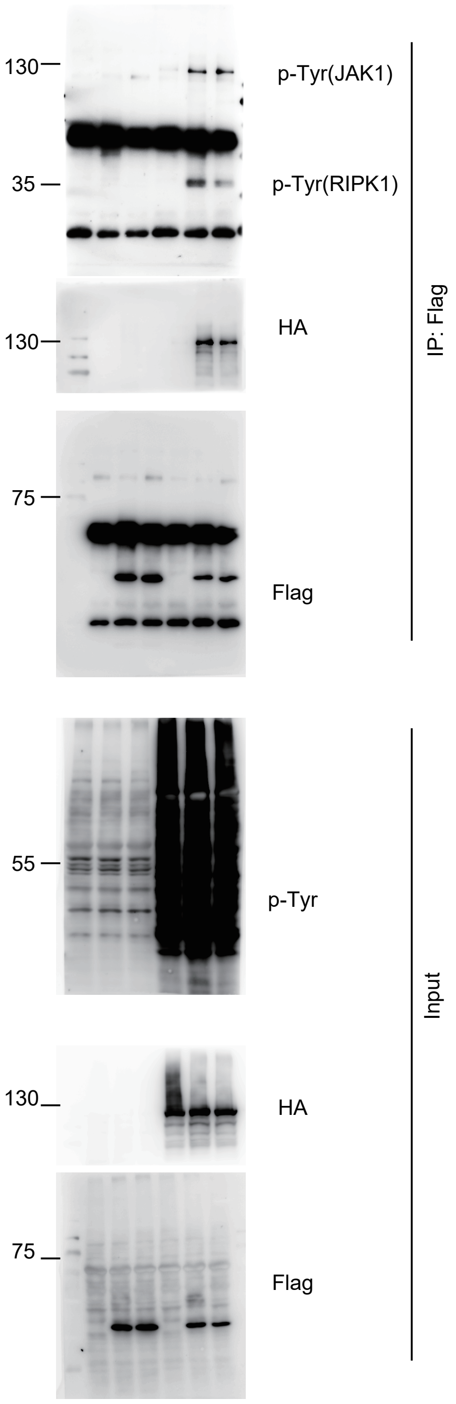

Figure 2g

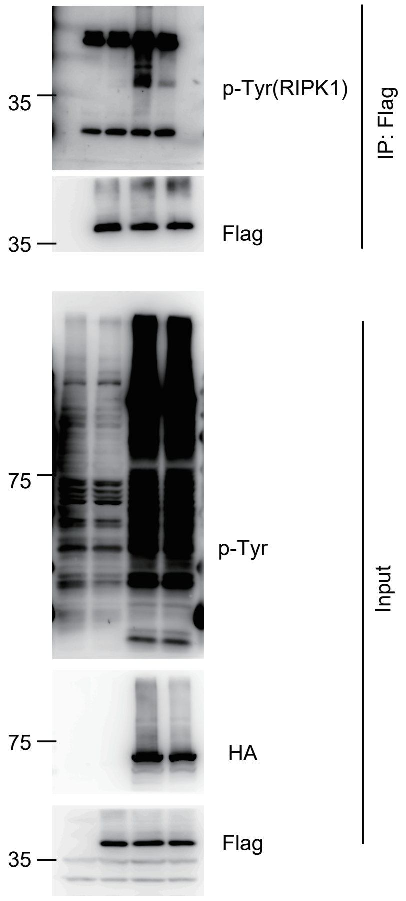

Figure 3c

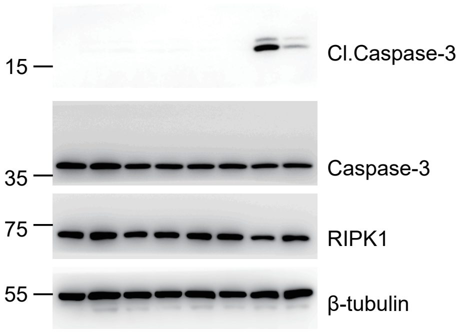

Figure 3e

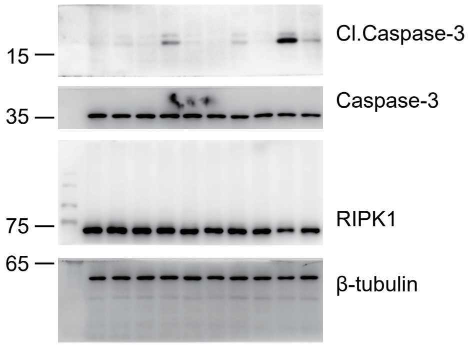

Figure 3f

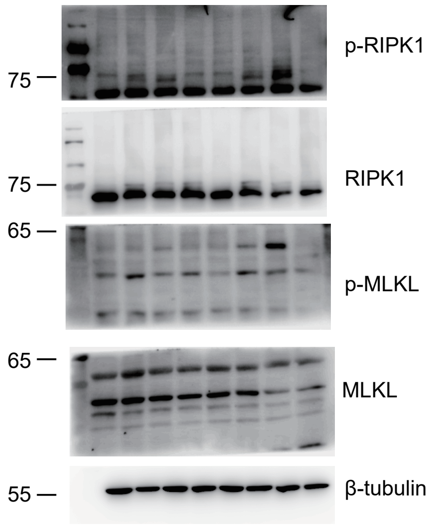

Figure 3g

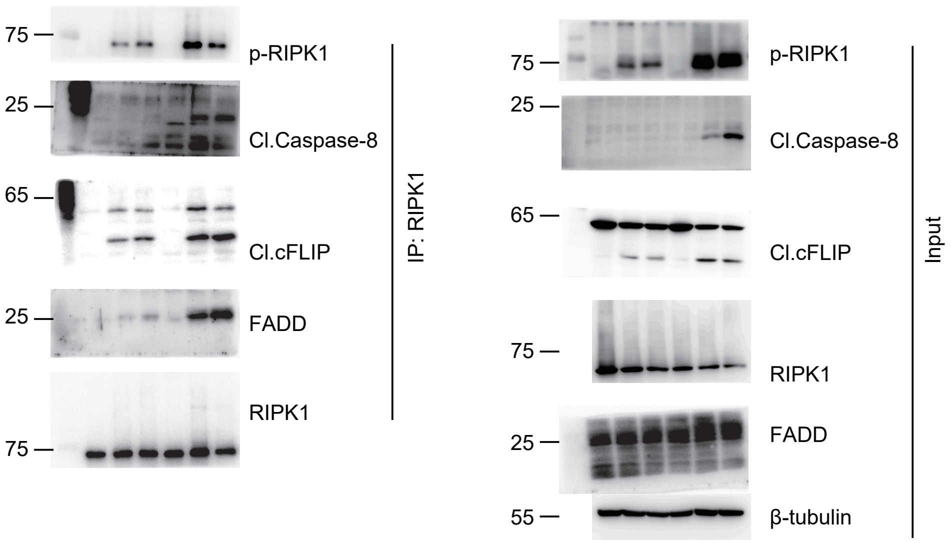

Figure 3h

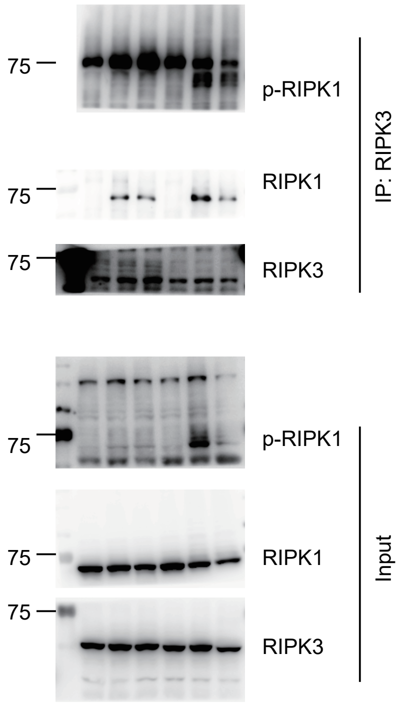

Figure 4a

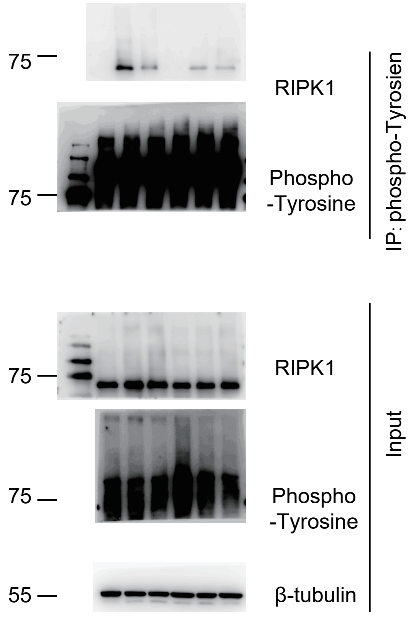

Figure 4b

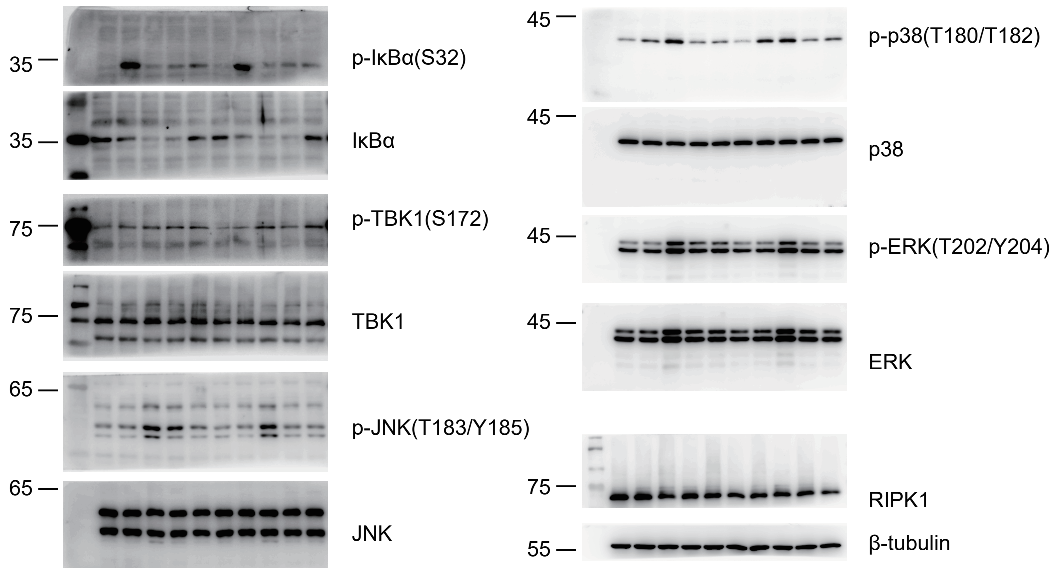

Figure 4c

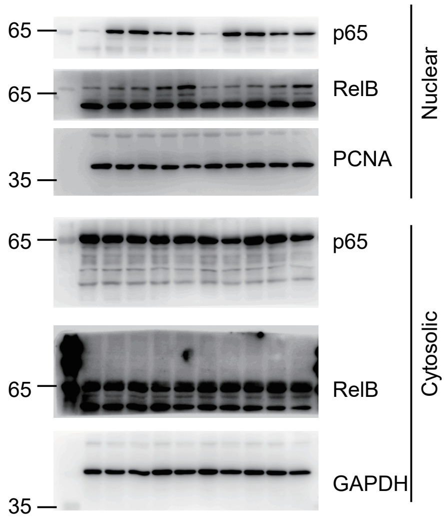

Figure 4e

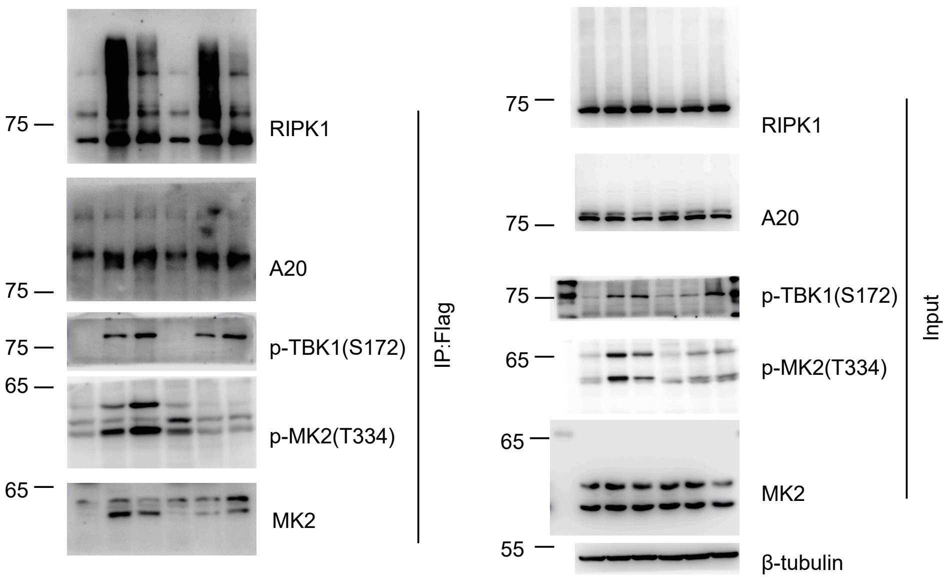

Figure 4f

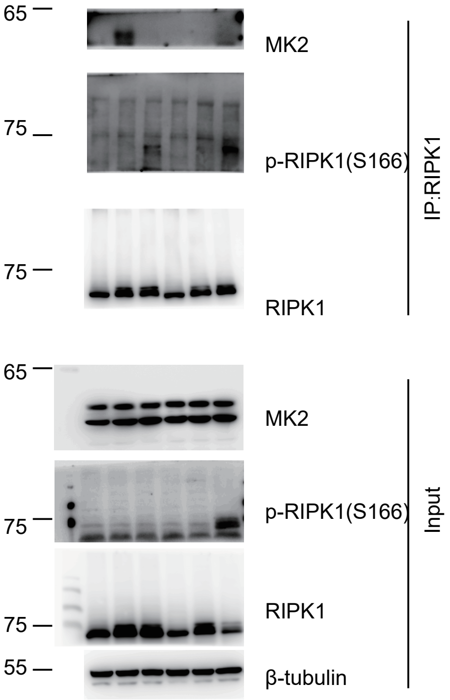

Figure 4g

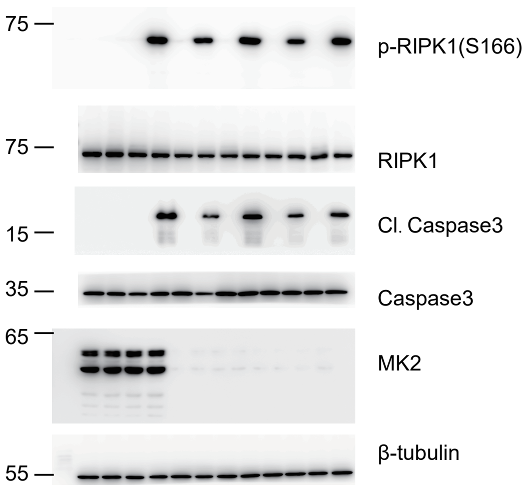

Figure 4i

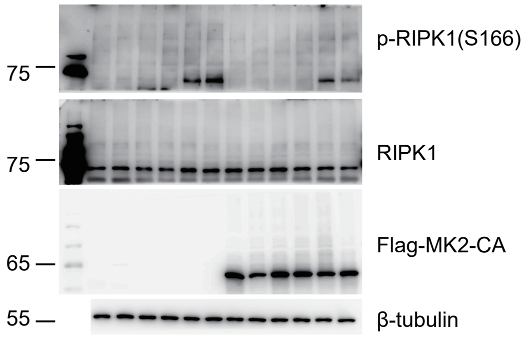

Figure S1b

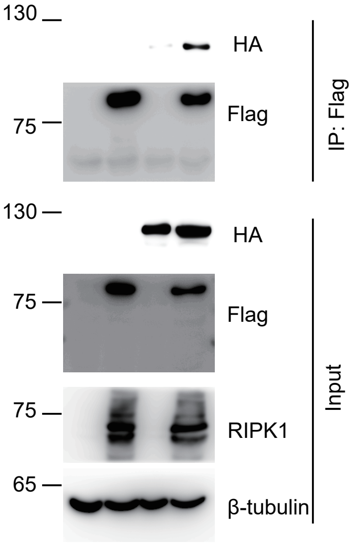

Figure S1c

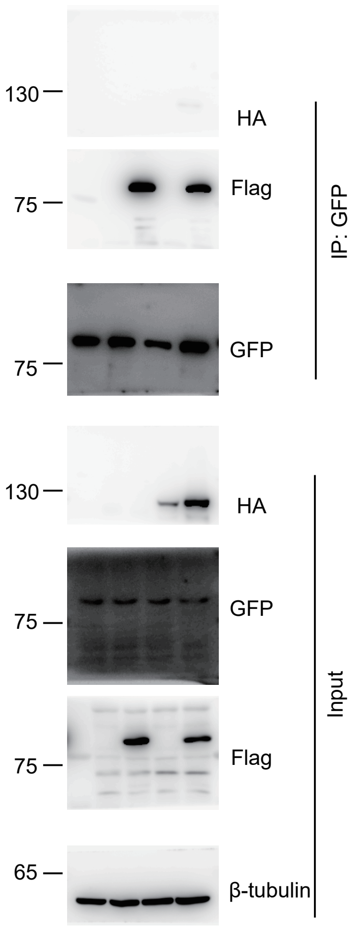

Figure S2a

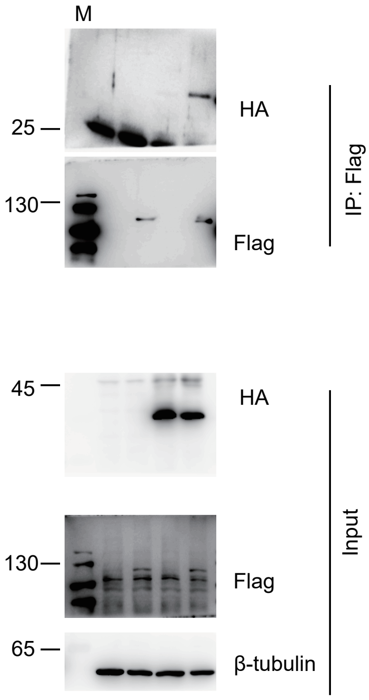

Figure S2b

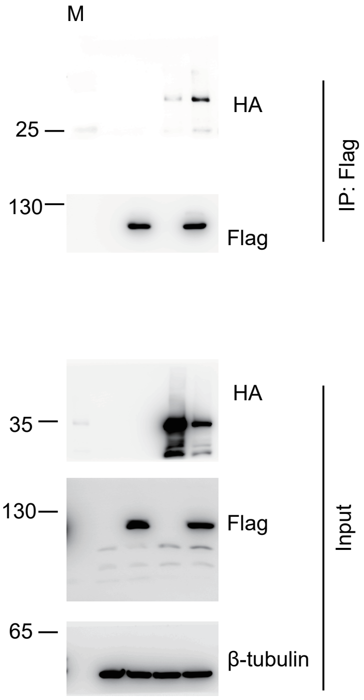

Figure S3f

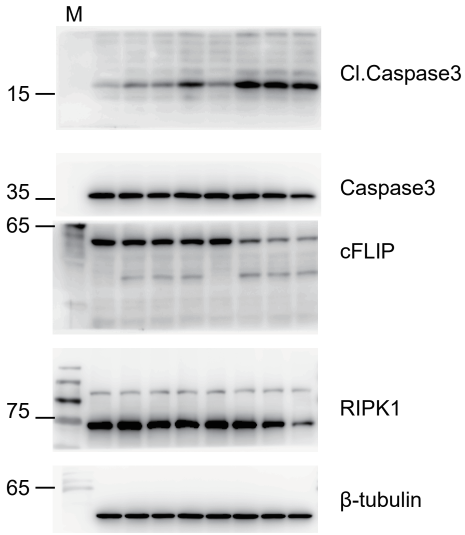

Figure S3h

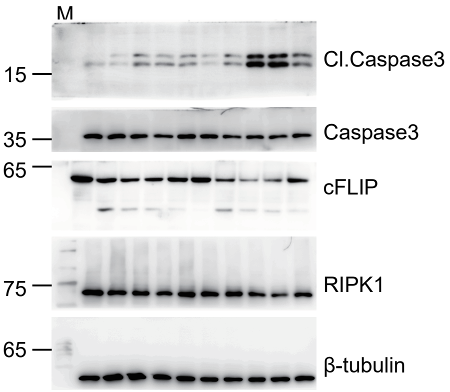

Figure S3i

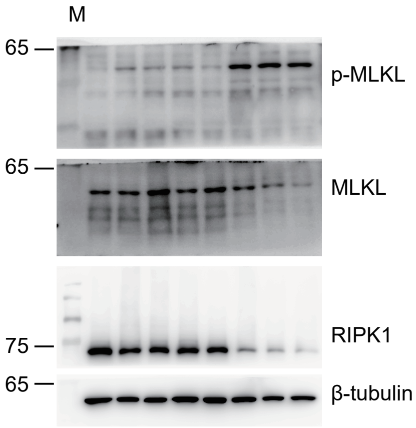

Figure S4a

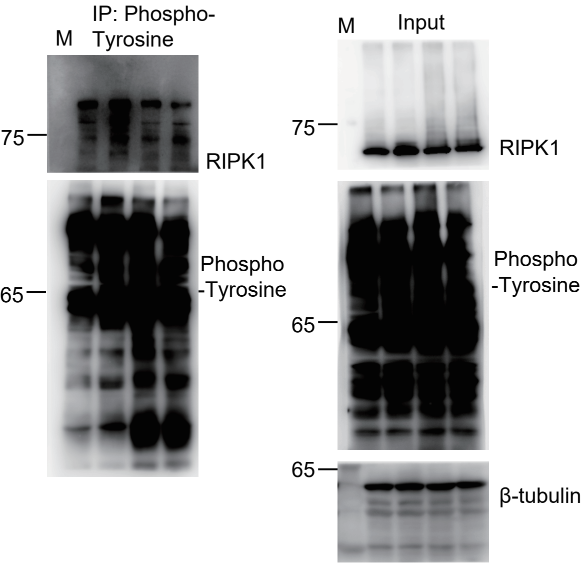

Figure S4b

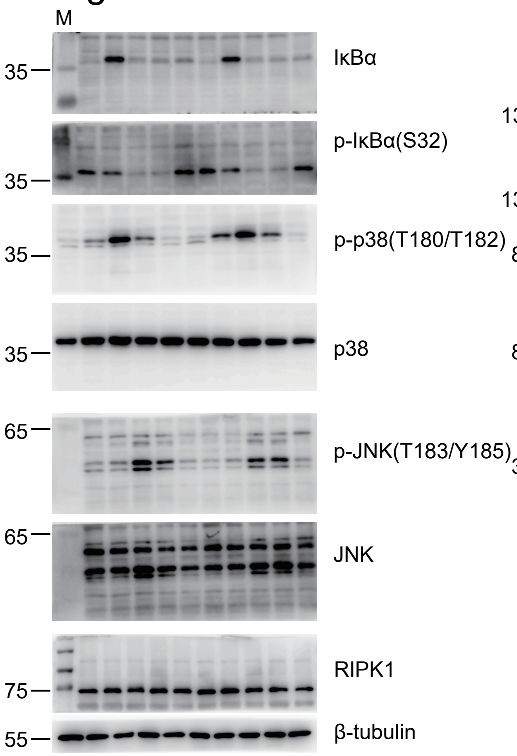

Figure S4d

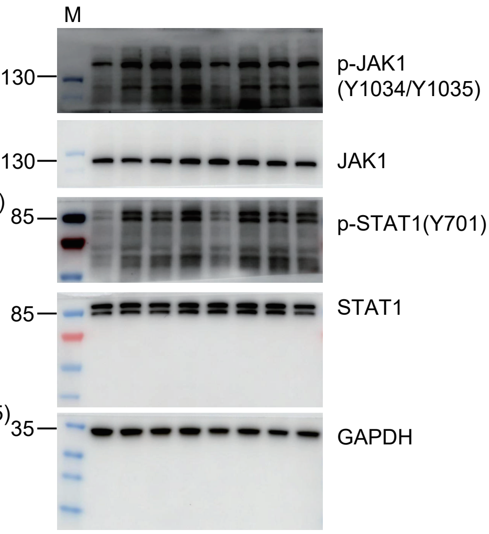

Figure S4e

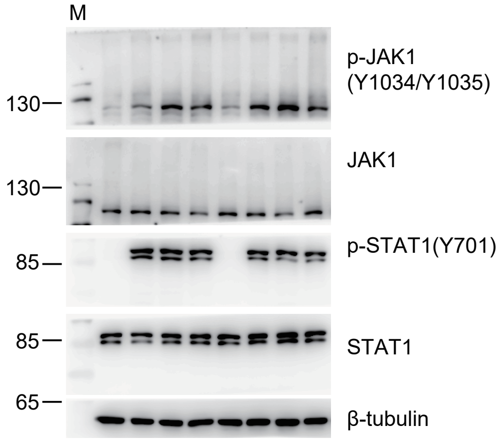

Figure S4f

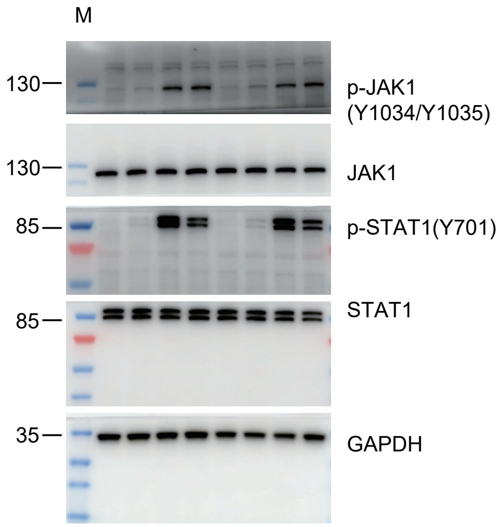

Figure S4h

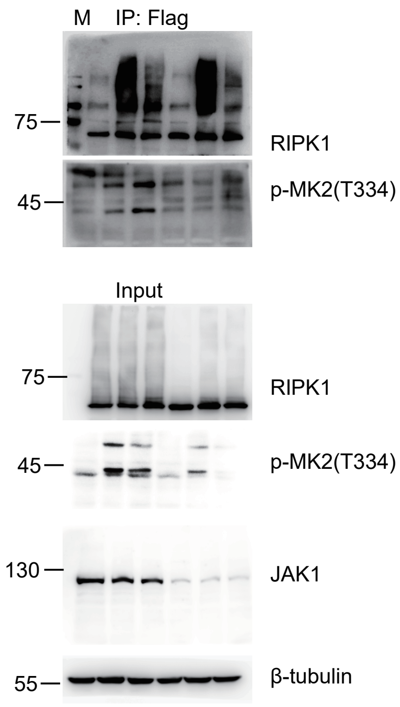

Figure S4i

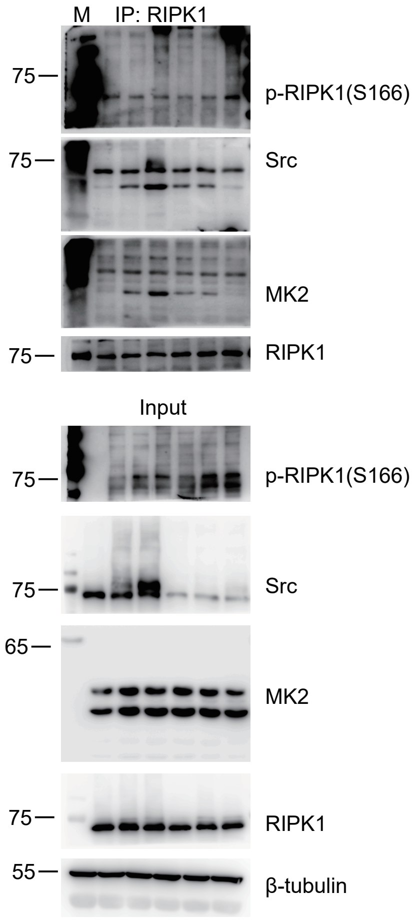

Supplement: Supplementary file 4 — Source Data [file 41467_2022_34080_MOESM4_ESM.zip › Source Data File 2.pdf]
