# Supplementary material for: Tyrosine phosphorylation regulates RIPK1 activity to limit cell death and inflammation
Source: Nat Commun. 2022 Nov 3;13:6603. doi: 10.1038/s41467-022-34080-4 (PMC9632600; doi:10.1038/s41467-022-34080-4)
Supplement: Supplementary file 4 — Source Data [file 41467_2022_34080_MOESM4_ESM.zip › Source Data File 3.pdf]

Uniprot ID	Gene	Score	Peptides	PSM
Q13546	RIPK1	10857.70664	63	2674
P0DMV8	HSPA1A	4003.58156	56	1061
P07900	HSP90AA1	1474.315216	66	409
P08238	HSP90AB1	1369.253247	61	393
E9PK54	HSPA8	913.9406958	18	276
P08670	VIM	753.4523443	45	204
P54652	HSPA2	397.0747018	20	135
Q14790	CASP8	467.9889833	33	131
Q16543	CDC37	379.8169526	25	118
Q13158	FADD	536.780224	15	109
O14654	IRS4	382.2829415	46	102
F5H265	UBC	254.0993223	11	99
P52272	HNRNPM	276.7234862	36	91
A0A0D9SF54	SPTAN1	269.2711312	62	79
E7EQ06	CASP8	241.1187774	21	76
Q01082	SPTBN1	229.1776898	47	62
M0R2S1	UBA52	151.4459826	8	60
E9PDF6	MYO1B	199.8615996	41	59
K7EL68	CDC37	175.6283481	10	53
P55072	VCP	159.7048298	34	51
Q13200	PSMD2	160.3123245	32	50
Q16643	DBN1	168.0108362	20	45
Q99615	DNAJC7	139.6686476	29	44
P31689	DNAJA1	155.2640878	18	42
P35998	PSMC2	119.706616	29	41
B4DEI6	MIB2	150.7972784	26	40
Q9UL15	BAG5	159.3016059	24	39
R4GNH3	PSMC3	147.4114577	24	38
A0A2P9AAP7	dnaK	136.7236402	3	36
P43686	PSMC4	118.1952699	19	32
O43242	PSMD3	89.13818038	23	32
Q15750	TAB1	125.4384956	20	32
Q12933	TRAF2	108.5226486	17	32
O00231	PSMD11	90.02152824	21	31
O94832	MYO1D	87.65551972	22	31
Q14126	DSG2	121.8820896	20	31
Q00610	CLTC	102.0243598	25	30
A0A024R254	MAGED1	100.3251613	19	29
Q96IR1	RPS4X	76.57729161	16	29
A0A024R201	PSMD13	72.41781676	18	28
O00232	PSMD12	92.60553253	20	27
Q9Y230	RUVBL2	83.4216187	20	27
P62195	PSMC5	90.17626786	17	26
P62333	PSMC6	82.76988518	16	26
Q96CS3	FAF2	103.1882929	12	25
Q9NYJ8	TAB2	92.37025094	16	24
P62191	PSMC1	76.16676581	13	24
A0A0A0MRM8	MYO6	78.93956959	20	24
B4E1Q4	RIOK3	69.43752849	14	24
E9PMH5	BIRC2	65.0806433	16	23
P62269	RPS18	56.95941281	14	23
P08754	GNAI3	82.90060794	11	23
E7EMC7	SQSTM1	91.06662154	10	22
E9PDE8	HSPA4L	74.29168999	17	21
P25685	DNAJB1	55.95533657	14	20
O60884	DNAJA2	66.20882225	12	20
Q9NVI7	ATAD3A	62.36101604	14	20
P20618	PSMB1	62.43930233	9	19

Uniprot ID	Gene	Score	Peptides	PSM
Q15008	PSMD6	46.0133512	13	19
A0A0S2Z3G5	CASP10	65.53593433	13	19
Q5CAQ5	TRA1	44.28600168	11	19
P04899	GNAI2	66.55808496	11	19
O00487	PSMD14	75.40698791	8	18
Q9Y4R8	TELO2	46.72602093	16	18
Q6P5Z2	PKN3	47.41948354	15	17
Q96CU9	FOXRED1	51.06529665	15	17
O43707	ACTN4	53.95999694	16	17
Q9NR30	DDX21	50.26233566	14	17
A0A0G2JL47	BAG6	60.97233677	13	16
Q86YT6	MIB1	59.60858142	11	16
Q15149	PLEC	40.59837091	16	16
P50402	EMD	64.11763358	10	16
Q9UHD2	TBK1	45.99430871	14	16
P28070	PSMB4	59.70467877	8	15
O43318	MAP3K7	40.95224547	12	15
Q15628	TRADD	60.63860822	9	15
P25789	PSMA4	44.07412934	9	14
Q3ZCQ8	TIMM50	53.63303447	6	14
P55036	PSMD4	47.18788791	10	13
P04792	HSPB1	55.02745426	8	13
P53618	COPB1	36.66765261	11	13
Q8IWV7	UBR1	39.08463752	11	12
Q92538	GBF1	29.50543535	9	12
O95071	UBR5	27.9149574	12	12
A0A0C4DGS1	DDOST	48.21752703	9	12
O15020	SPTBN2	34.51803446	11	12
Q5T9A4	ATAD3B	36.54270625	9	12
Q05DH1	PSMA7	39.60042	8	11
P25787	PSMA2	45.20817626	8	11
P28074	PSMB5	40.56315589	8	11
Q5W0B1	OBI1	33.97364056	8	11
P53621	COPA	33.30454803	10	11
A0A024R9Y3	HUWE1	35.03097939	11	11
A6NDG6	PGP	38.91283405	9	11
Q8IWV8	UBR2	34.61723089	8	10
A0A087WY00	HEATR1	28.21109247	10	10
C9J3G2	CBWD2	32.75912428	7	10
Q8IYV2	DDX20	31.99750483	9	9
P28066	PSMA5	29.27536964	7	9
G3V0E5	TFRC	19.68357778	8	9
K7EJ78	RPS15	34.13046658	3	9
Q8TAT6	NPLOC4	30.57419348	7	9
Q6PKG0	LARP1	24.28517175	8	9
Q04656	ATP7A	31.65534091	9	9
P46781	RPS9	17.09538066	8	9
Q96S55	WRNIP1	32.31487918	8	9
O75955	FLOT1	27.04532635	8	8
Q53GT1	KLHL22	23.4305377	8	8
H7C463	IMMT	29.27822638	7	8
P10398	ARAF	18.87548065	7	8
A0A024R0K2	CSDE1	20.07306588	8	8
B7ZMF2	FANCI	16.00529349	8	8
Q9UMS4	PRPF19	30.20177376	6	8
F8VNX8	TRAFD1	27.89334273	5	8
P25786	PSMA1	14.67609227	6	8
P07195	LDHB	16.40625107	8	8

Uniprot ID	Gene	Score	Peptides	PSM
H7BXI1	ESYT2	24.80228448	7	8
Q14683	SMC1A	24.72317028	8	8
P52701	MSH6	22.54397154	8	8
P62081	RPS7	26.68705821	7	8
C9JXB8	RPL24	20.34689713	5	8
I3L1P8	SLC25A11	23.78230762	8	8
P46778	RPL21	22.11653423	4	8
H7BXY3	DHX30	25.82035971	8	8
P60900	PSMA6	20.7657547	6	7
Q6FG43	FLOT2	22.37499905	7	7
A0A024R847	C7orf20	19.97774792	4	7
Q13573	SNW1	17.55042481	6	7
Q9BT22	ALG1	18.94651246	5	7
P21796	VDAC1	21.36447239	6	7
P10644	PRKAR1A	18.28473246	5	7
P49721	PSMB2	19.12394118	4	7
P08708	RPS17	22.94053769	4	7
Q71UM5	RPS27L	15.01434803	3	7
Q96BH1	RNF25	21.32512426	5	7
A0A024R2Z6	GNL3	21.99230599	7	7
Q96F88	POP1	19.7744627	6	7
M0QZR8	POLD1	17.17860067	6	7
B7ZM73	MON2	22.24184442	6	7
B4DNN2	DNAJB4	14.40762758	4	6
Q96AG4	LRRC59	18.46230173	5	6
Q9NW81	DMAC2	19.32931376	4	6
P25788	PSMA3	12.84098721	5	6
P48444	ARCN1	16.024611	6	6
Q96EP0	RNF31	15.86585522	6	6
P13010	XRCC5	13.9777503	6	6
P62854	RPS26	9.62977457	4	6
D3DWY0	IKBKG	21.93439126	5	6
P62851	RPS25	15.1771524	5	6
A0A1W2PRV5	SMN2	19.77928162	4	6
A6QKW0	SHINC3	14.86814499	5	6
P62753	RPS6	15.17971039	4	6
K7EMW4	NCLN	18.85274577	6	6
Q6P4A7	SFXN4	14.95504236	4	6
Q9C0C2	TNKS1BP1	24.03194857	5	6
P28072	PSMB6	23.23891377	4	5
Q9BYN8	MRPS26	14.15795958	4	5
Q9Y512	SAMM50	15.89257252	5	5
Q16186	ADRM1	7.474870443	5	5
Q9NXS2	QPCTL	14.85311234	4	5
Q15070	OXA1L	10.24505126	5	5
Q96T17	MAP7D2	17.85090971	4	5
A0A0M3R6J9	MAP7D2	17.8890059	4	5
Q2KHR3	QSER1	19.16621721	5	5
Q13535	ATR	11.77981997	5	5
O75643	SNRNP200	18.05520988	4	5
E7EMV7	TNIP1	12.71938539	5	5
Q9ULX6	AKAP8L	15.84999084	5	5
G3XAM7	CTNNA1	16.89828706	4	5
P35606	COPB2	17.33225346	5	5
A0JP11	PIK3R4	12.41704154	5	5
A0A024R7L5	UPF1	15.40688157	4	5
P29966	MARCKS	27.84936047	3	5
P46783	RPS10	14.74123406	3	5

Uniprot ID	Gene	Score	Peptides	PSM
P12277	CKB	17.69673324	5	5
Q7Z4H7	HAUS6	16.30770469	5	5
P04181	OAT	13.72277045	5	5
D6REM6	MATR3	22.9609077	4	5
O75155	CAND2	15.88652039	5	5
A0A087WTG3	CUL3	13.19649279	2	5
P12931	Src	4.57301259	4	5
H0YGQ5	ARMC6	7.895508528	3	4
Q9HBH1	PDF	15.04046321	3	4
R4GMR5	PSMD8	14.14268041	4	4
H3BLV0	CD55	11.388008	4	4
O75190	DNAJB6	9.799087763	4	4
Q5TB53	TM9SF3	12.41786063	3	4
Q9H974	QTRT2	13.30856192	4	4
O75953	DNAJB5	10.0210954	4	4
A0A087WT84	JMJD4	14.49146938	3	4
O95817	DKFZp686H09231	10.25034618	3	4
Q14677	CLINT1	13.32847619	4	4
J3KPS2	FAM83H	13.95037031	4	4
I3L0J9	PRPF8	12.52287686	4	4
D3DUJ0	AFG3L2	10.95330548	4	4
O94822	LTN1	11.51632929	4	4
A6H8X9	CEP170	11.7111845	4	4
D6R939	IQGAP2	9.927880526	1	4
H7C2W9	RPL31	14.88599563	3	4
H7C1G6	ATG9A	15.23868537	3	4
P42766	RPL35	7.836481571	3	4
Q9UJZ1	STOML2	15.77673602	4	4
B5MC98	PREB	16.28563404	3	4
Q9UNE7	STUB1	11.91924429	4	4
A0A024R1J3	CDC42EP1	15.23715305	3	4
G9FP35	GNAQ	8.877077818	4	4
P07477	PRSS1	12.74451756	2	4
Q9Y6A5	TACC3	18.7927916	4	4
Q5VV42	CDKAL1	13.25914359	4	4
P50395	GDI2	12.68712878	3	4
Q15758	SLC1A5	11.53648722	3	4
Q01813	PFKP	10.23236489	4	4
B4DIP2	ERBIN	11.56778646	3	4
B8QGS9	PKP2	13.96853828	3	4
Q9NZQ3	NCKIPSD	9.474038601	4	4
Q14573	ITPR3	9.973967314	4	4
Q5JRU2	CASP9	12.89848781	3	3
H7C3I1	ST13	9.307614565	3	3
Q9Y3U8	RPL36	4.329216003	3	3
J3QL04	ASPSCR1	12.37751722	3	3
H0YL12	ETFA	9.101257563	3	3
O43819	SCO2	10.32090735	2	3
H0YEE1	VPS51	7.182109714	3	3
Q9HC07	TMEM165	14.64630747	2	3
C9JYS8	NONO	4.76258409	3	3
Q15717	ELAVL1	7.921278477	3	3
P04040	CAT	11.03011131	3	3
Q96P63	SERPINB12	10.77790082	3	3
Q9NQ51	FTSH	10.25624609	3	3
A0A0C4DGG1	PACSIN3	7.844673634	3	3
Q9NZ09	UBAP1	9.759977818	3	3
Q13151	HNRNPA0	8.97133553	2	3

Uniprot ID	Gene	Score	Peptides	PSM
B3KW56	EIF3E	7.428546429	3	3
P78368	CSNK1G2	6.377847075	3	3
Q9UPY6	WASF3	11.89073467	2	3
Q08AM6	VAC14	8.869672537	3	3
Q96G38	EIF3B	10.05334115	3	3
Q5SY16	NOL9	2.028900862	3	3
Q3LRJ5	NBR1	5.006340504	3	3
Q13049	TRIM32	10.06753898	3	3
Q05BS0	EIF3A	7.366168261	3	3
Q8IXI1	RHOT2	6.786955714	3	3
O94887	FARP2	12.82978439	2	3
O00291	HIP1	8.185810208	3	3
A1L3A9	TBC1D9B	10.22028279	3	3
O15397	IPO8	7.295622826	3	3
H0YHR8	NT5C2	10.87107825	2	3
A0A024R755	CALU	10.49895263	3	3
Q15404	RSU1	8.525016904	3	3
A0A097Q0T5	COX2	5.771396041	3	3
E7EQB9	POLR1C	10.06814587	3	3
E9PK01	EEF1D	5.576726198	3	3
D3DR40	C10orf4	9.367878199	3	3
B4DFY5	MAP2K1	7.84365356	3	3
C9J3L8	SSR1	7.008049607	3	3
Q7Z624	CAMKMT	6.722880244	3	3
Q6PJT4	MSN	7.377729177	3	3
Q8WWH5	TRUB1	7.56784749	3	3
U3KQC1	WDR18	6.510646343	3	3
A0A024RC87	RNH1	7.976518512	3	3
Q05CN7	GTF3C4	7.154960155	3	3
Q05DU1	GNL3L	7.549282074	3	3
B7ZMD6	IRGQ	7.244283199	3	3
Q7RTQ9	KNS2	9.429313183	3	3
P46087	NOP2	7.179776907	3	3
A0A2R8Y695	IQSEC1	11.6828773	3	3
Q9UPN7	PPP6R1	9.633641243	3	3
Q9BZQ6	EDEM3	9.291030169	3	3
Q16513	PKN2	8.341866732	3	3
P07942	LAMB1	11.25534248	3	3
E9PPJ1	KNL1	4.806189179	3	3
Q05CF5	CFHR5	4.140742779	1	3
Q14204	DYNC1H1	7.020519137	3	3
B3KW74	PHGDHL1	5.490026712	2	2
C9JSU3	CFLAR	5.635615945	2	2
F8WEG8	PRKRA	4.773425579	2	2
J7HBC9	RAET1H	4.424120903	2	2
M0R001	COQ8B	10.83837795	2	2
K7ELQ0	MRPL4	3.965979099	2	2
A0A087X283	UBE2E2	3.512315154	2	2
H0YDS2	TRMT44	9.83037281	2	2
Q14CB2	DOK1	9.219404221	2	2
A2VCR0	MAPRE1	6.320755243	2	2
I3L3P7	RPS15A	4.226703644	2	2
Q5T8U9	CARNMT1	7.105725288	2	2
M0R0P4	STRN4	5.849295139	2	2
G3V2M8	SCFD1	8.034205198	2	2
C9JDH9	STK25	4.071372509	2	2
B0QYW5	SLC25A17	7.294696093	2	2
B4DIH5	COPS2	7.501613379	2	2

Uniprot ID	Gene	Score	Peptides	PSM
H0Y715	CACNA2D1	9.666369438	2	2
A0A0U1RQQ4	PROCR	6.283441067	2	2
J3QT95	COPS7B	7.818271875	1	2
P51970	NDUFA8	4.368692756	2	2
Q9BS14	GANAB	5.739869595	2	2
E9PQU5	RBM25	2.365814447	2	2
Q8WUK0	PTPMT1	3.34973526	2	2
E9PFP1	PIK3R2	7.479541063	2	2
P28289	TMOD1	6.503740311	2	2
F5H4V9	PDCD2	6.079699755	2	2
Q96NB2	SFXN2	6.040197611	2	2
A0A024R9T1	hCG_39634	8.465930939	1	2
A0A0S2Z565	HAX1	4.764981747	2	2
A0A024R588	SF1	4.208080769	2	2
A0PJK4	NUP43	5.472508907	2	2
Q2NLD4	PURA	8.991302729	1	2
A0A0S2Z5B8	DCTN4	7.031147003	1	2
Q14249	ENDOG	4.931644678	1	2
Q7KZN9	COX15	4.206242085	2	2
A6NHG8	TRAF3	4.291200638	2	2
Q96FA6	NCAPD3	6.94415164	2	2
A0A0C4DG44	HTRA2	4.597696662	2	2
A0A024R863	MAPKAP1	5.806300879	2	2
F6UYG0	WNK1	5.20597434	2	2
Q96A33	CCDC47	7.232571363	2	2
Q969U6	FBXW5	7.000089645	2	2
Q05655	PRKCD	6.521875143	2	2
Q8IYI6	EXOC8	3.015182972	2	2
B2R894	MRPL38	4.562377453	2	2
Q5VZM0	RRAGB	4.444955349	1	2
Q8NHP8	PLBD2	1.870266557	2	2
A0A087WW88	CHMP2B	4.006007671	2	2
Q9BVI4	NOC4L	5.683113337	2	2
A8DPD7	ZYG11B	5.241337776	2	2
H7BZQ3	OTUD5	9.027451038	1	2
Q96PK6	RBM14	5.037018776	2	2
Q969S3	ZNF622	6.188467026	1	2
A0A087WW77	LLGL1	5.777001619	2	2
Q6PK82	AP3D1	9.461499929	1	2
E7ENQ1	MAP4K4	5.433651447	2	2
Q12860	CNTN1	1.734054327	2	2
A0A0A0MRP0	MAP7D3	3.973709702	2	2
Q9P2L0	WDR35	3.417207718	2	2
Q96JB3	HIC2	5.562573671	1	2
P46939	UTRN	5.878582001	2	2
Q92621	NUP205	2.330450058	2	2
Q9Y6V0	PCLO	3.480222344	2	2
P49137	MAPKAPK2	5.679451466	2	2
Q4LDX3	JAK1	2.064415693	2	2
H0YJ55	ATP6V1D	2.778212309	1	1
A0A1W2PRU5	OSBPL1A	2.948906183	1	1
E9PKZ9	TMEM126B	2.523631096	1	1
F8WBC0	RAP1B	2.220353365	1	1
C9JL85	MTPN	4.862388611	1	1
C9JGA2	AZI2	6.101532936	1	1
K7EPW5	PJA1	2.997603893	1	1
E9PQK5	RRAS2	2.037054062	1	1
Q8WUH6	TMEM263	3.664801359	1	1

Uniprot ID	Gene	Score	Peptides	PSM
E9PJW1	STX17	2.4962883	1	1
E9PKL8	NDUFS3	3.642196417	1	1
V9GYN3	HSD17B7	3.15560174	1	1
I3L1T3	DERL2	4.875636578	1	1
A0A087WUM5	GLIPR2	2.859193563	1	1
K7EN82	NMT1	2.812577724	1	1
F5H4I5	ITM2C	4.987983704	1	1
F8WEF0	ATIC	3.137937546	1	1
E9PPB0	ZNF143	4.764967442	1	1
F8WEN7	MTFP1	2.020722151	1	1
D6RF21	MTRR	2.704828024	1	1
Q96IX5	ATP5MK	2.036121368	1	1
E5RHY8	MRNIP	3.595995188	1	1
F8WD66	CCZ1B	3.340000153	1	1
H0YDV6	DENND5A	4.565376282	1	1
A0A087X1A9	SEC22B	3.249600172	1	1
M0QYL4	AKAP8	2.505926132	1	1
Q8IZV2	CMTM8	3.469573259	1	1
H0YCU2	SHARPIN	2.565279961	1	1
J3QQY2	TMCO1	3.136112452	1	1
V9GYZ1	ITGB5	2.127091408	1	1
A0A087X030	NEK3	2.78799367	1	1
F2Z3I9	CASTOR1	1.687974691	1	1
B4DHN5	SDCBP	6.652760983	1	1
H0YII4	DDI2	2.318508387	1	1
A0A1D8GZE1	PIK3R1	4.246486664	1	1
A0A2U3TZN1	SRP19	2.736566544	1	1
Q9H061	TMEM126A	3.235903502	1	1
Q0VDC6	FKBP1A	3.272917747	1	1
K7ERQ7	SPC24	2.865931273	1	1
K7EJ44	PFN1	2.821806908	1	1
E5RI90	C1orf198	4.720580101	1	1
H0YDD8	RPLP2	2.575507641	1	1
E5RHM7	TTI2	3.275511742	1	1
P61956	SUMO2	2.216846466	1	1
H7C0U0	PARL	3.377846003	1	1
H0YCE7	FIBP	2.459820271	1	1
P82930	MRPS34	4.152692795	1	1
K7EKF3	ERCC2	4.635057449	1	1
B1AR62	HK1	2.589398861	1	1
H3BMT0	JPT2	2.029349804	1	1
A0A024R9M5	CEP27	3.667411566	1	1
P17677	GAP43	3.955074072	1	1
F8VQF4	CNOT2	2.786365747	1	1
Q8N1B9	NDUFA10	2.558079481	1	1
C9JYM0	POP7	2.415754557	1	1
H0YEY4	NUDT5	2.852278709	1	1
M0R1B5	ILVBL	4.414130688	1	1
A0A024R187	LOC150223	3.19311285	1	1
F5H6J5	ZCCHC8	3.658570051	1	1
B7Z8H2	TRDMT1	2.002222061	1	1
K7ERN7	NLE1	3.176728487	1	1
E9PIQ8	BAG3	2.640636921	1	1
B4DTT8	STIM1	6.609516621	1	1
H0YGX8	COG6	2.302153111	1	1
G1UD80	SPACIA2	4.543738365	1	1
A0A2P9AUT8	yfbT	2.427487612	1	1
H7C4L3	SKIV2L	3.100589991	1	1

Uniprot ID	Gene	Score	Peptides	PSM
E5RGR9	YIPF5	2.003448009	1	1
Q9Y241	HIGD1A	1.863965511	1	1
K7ERG2	NKIRAS2	1.814808249	1	1
K7ELV2	SEH1L	4.272314072	1	1
H0YIZ6	HAUS4	3.27321744	1	1
H0Y4J2	MRPL37	3.930133343	1	1
E9PRZ9	C11orf58	1.627031207	1	1
Q6Q4G9	NEU1	2.09746623	1	1
Q5JXM0	DKFZp564C0482	2.906029701	1	1
B1AHF3	CYB5R3	3.434898376	1	1
E5RJP2	SORBS3	2.426464319	1	1
C9J6N9	UFD1	2.965882063	1	1
Q6P6C2	ALKBH5	4.429862976	1	1
H0YCA5	SPATA5L1	4.20899868	1	1
Q7Z777	CLPB	2.814167261	1	1
B4E1G1	DERL1	2.39676404	1	1
B0QYP8	PARVB	4.158995628	1	1
Q5T9P8	PRPF18	2.157859802	1	1
H0Y4S7	RBCK1	4.173136135	1	1
D3TTZ0	TNFAIP3	4.372313023	1	1
E9PE17	MRPS17	2.293159485	1	1
E9PKA7	RCE1	2.653888226	1	1
Q96SX4	GTPBP3	3.332459211	1	1
P19387	POLR2C	4.032269955	1	1
A0A024R1S6	UNC84B	3.279503584	1	1
C9JJK5	ZYX	2.415780067	1	1
F8WF02	PDHB	3.655051231	1	1
B7Z8W6	NAMPT	3.386403799	1	1
H0YB37	TTC1	2.079128981	1	1
Q96F63	CCDC97	4.396150589	1	1
A0A0A0MSZ9	CSTF1	3.851165533	1	1
H0Y783	FARP1	3.355931997	1	1
Q8N0V3	RBFA	5.040665627	1	1
A0A024RAP0	XRCC4	4.162993431	1	1
Q659F8	DKFZp434F152	2.640876532	1	1
P23258	TUBG1	6.550103664	1	1
Q92522	H1-10	2.17494607	1	1
Q86W42	THOC6	2.950344563	1	1
Q53SY8	GTF3C2	2.592338562	1	1
H7BZR7	DGKD	2.252041817	1	1
Q712K3	UBE2R2	2.180266619	1	1
S4R328	IGF2R	2.218544245	1	1
Q6PJ70	OSBPL6	2.771759987	1	1
A0A0U3FJG0	CXCR4	2.999687195	1	1
P48047	ATP5PO	2.061857462	1	1
A0A2P9ANE5	BQ8482_300050	2.070933342	1	1
Q9UFV9	DKFZp434J046	3.223028183	1	1
Q9H384	TCF25	5.475764751	1	1
A2NFR5	Hin-3	4.178281784	1	1
Q96KC2	ARL5B	2.65951252	1	1
E9PN39	MTFR2	3.209722519	1	1
Q8TDN6	BRIX1	2.709032536	1	1
Q53ET0	CRTC2	6.374731541	1	1
Q8N0Z8	PUSL1	2.22067976	1	1
G5EA42	TMOD2	3.882229805	1	1
Q8NBK3	SUMF1	2.582529545	1	1
Q8NDD3	DKFZp586M1819	3.398692846	1	1
Q9P0J7	KCMF1	4.451453209	1	1

Uniprot ID	Gene	Score	Peptides	PSM
G3V4Q6	PRKCH	2.742133379	1	1
Q5T0Y8	SMPDL3B	3.798241615	1	1
Q6PD74	AAGAB	2.669812918	1	1
H7C0X5	CPVL	2.044848442	1	1
H7C0V0	MAIP1	2.908654213	1	1
H0YGW8	DUS1L	3.264554739	1	1
G3V4F7	SRP54	3.418725967	1	1
Q9NPD3	EXOSC4	2.290134192	1	1
B1AK81	PIGK	2.892471552	1	1
A0JLU2	BUB1	4.326336384	1	1
Q8IYU8	MICU2	4.541089535	1	1
S4R446	MEX3D	2.88275218	1	1
Q8TBP6	SLC25A40	3.110319138	1	1
A0A024R500	RP3-402G11.12	2.737948656	1	1
O75486	SUPT3H	2.240816355	1	1
Q9UHR4	BAIAP2L1	5.282747746	1	1
A8K7S5	KATNA1	3.100812435	1	1
Q63HP7	DKFZp686P1551	3.639596462	1	1
Q9H4K1	RIBC2	2.031704664	1	1
Q01650	SLC7A5	3.489608765	1	1
A0A0B4J2G0	ZNF107	2.615180731	1	1
J3QSX6	ABLIM1	2.774361849	1	1
Q8TAR2	EXOC4	2.700289726	1	1
E9PC44	SEC24D	2.455662489	1	1
F6SS63	TAB3	3.701022148	1	1
Q9BVR6	TUBGCP4	2.158443689	1	1
Q53SG7	HK2	3.103965521	1	1
A0A2P9ADJ3	hisS	2.877591133	1	1
A0PJA5	GCC2	2.442413568	1	1
Q5LJB0	UCHL5	2.139441013	1	1
Q5T1M7	PRPF4	3.517625809	1	1
A0A0J9YWQ2	PTDSS2	2.413752317	1	1
A0A2R8Y6T6	EPB41	1.822489977	1	1
A0A2P9ADQ2	BQ8482_111181	1.772714496	1	1
X5D7P8	STK39	3.672755718	1	1
Q9BTE2	C16orf35	1.744378448	1	1
Q9Y3W7	DKFZp586J0119	2.582301378	1	1
D3DV11	FAM63A	2.614818811	1	1
A0A087X2C4	PCDH7	3.476895094	1	1
A0A2P9AE76	BQ8482_111364	2.025850058	1	1
Q13077	TRAF1	2.517351627	1	1
Q9H7Z6	KAT8	2.623185158	1	1
Q05BU5	RBM16	1.684382558	1	1
A0A024RCU9	SRPK1	2.796896458	1	1
H0YFS7	SALL2	4.811727524	1	1
Q96ET8	TVP23C	1.615409136	1	1
J3KN66	TOR1AIP1	3.00462532	1	1
C9JVY5	PALM2AKAP2	4.502130032	1	1
A0A2P9AK41	BQ8482_20119	2.497256756	1	1
K7EP32	UBXN6	2.132875919	1	1
Q9H6T3	RPAP3	2.916704416	1	1
Q9UBV2	SEL1L	3.188847303	1	1
Q9UBI9	HECA	3.528794289	1	1
Q9Y5E9	PCDHB14	3.513257742	1	1
A0A2P9AS54	BQ8482_380171	3.042038679	1	1
Q7L9B9	EEPD1	1.958341122	1	1
Q6UVY6	MOXD1	3.194591999	1	1
Q13325	IFIT5	3.054144621	1	1

Uniprot ID	Gene	Score	Peptides	PSM
Q5W0U4	BSPRY	2.217218637	1	1
Q2TBD1	NEBL	2.141103983	1	1
P23677	ITPKA	2.422858	1	1
A0A024R035	C9	2.391150951	1	1
Q96D09	GPRASP2	4.1456604	1	1
B9EG90	TOP1	2.859921455	1	1
O94972	TRIM37	4.601671219	1	1
Q9NVV4	MTPAP	2.702844143	1	1
P10155	RO60	2.586267948	1	1
Q6P2P2	PRMT9	3.042501688	1	1
B7Z685	GUCY1B1	2.302767754	1	1
H0Y8V8	ZCCHC4	2.194715261	1	1
A0A2R8YCL0	SERAC1	2.320869923	1	1
Q2TBE0	CWF19L2	2.139574766	1	1
Q86YS3	RAB11FIP4	2.821898937	1	1
A5D6X0	SMARCA4	2.331985474	1	1
Q9NX84	EPB41L4B	1.986981392	1	1
O95972	BMP15	1.799469233	1	1
Q4G0A6	MINDY4	1.749332547	1	1
Q8N5Z0	AADAT	2.087830782	1	1
Q03252	LMNB2	2.509961367	1	1
Q12788	TBL3	3.274035692	1	1
H7BZJ7	FANCD2	2.459842443	1	1
U5Y1J8	SP140L	1.849817753	1	1
Q9H4H8	FAM83D	1.977354169	1	1
Q9ULE6	PALD1	2.889243364	1	1
A0A2P9AHQ9	BQ8482_170031	1.892455339	1	1
A0JNW5	UHRF1BP1L	2.316401243	1	1
C9J8U1	SPECC1L	2.394709587	1	1
A2A3K4	PTPDC1	2.487509489	1	1
B1ALB4	SMG7	1.980963349	1	1
Q5THK1	PRR14L	4.701679707	1	1
D6RCC7	FAM135A	2.091178656	1	1
Q8N6J1	BTAF1	2.096361637	1	1
E9PMP7	LMO7	2.922794819	1	1
Q9H8M5	CNNM2	1.934566498	1	1
O43379	WDR62	2.290194511	1	1
Q5T795	MDN1	2.762516499	1	1
H7C5W6	HEATR5A	4.730481625	1	1
A0A1V1G0D9	TSC2	2.480282307	1	1
D1CS68	TLR7	2.374367237	1	1
A0A024QZW7	NUP153	2.85513401	1	1
P14543	NID1	2.232343435	1	1
Q5EBL9	PLEKHG1	2.06656909	1	1
A0A087WW76	ARHGAP21	3.231390953	1	1
F8VRX1	RAPGEF3	2.328705549	1	1
A0A0A0MS99	ABCC1	2.125915051	1	1
H0YME5	EIF2AK4	2.477323532	1	1
A6NGG8	PCARE	1.976334333	1	1
Q96BY7	ATG2B	2.19435811	1	1
B2RU27	TEX15	2.085278034	1	1

Red: Known RIPK1 regulators; Blue: genes with interest
